# Chloroplast genomes of *Byrsonima* species (Malpighiaceae): comparative analysis and screening of high divergence sequences

**DOI:** 10.1038/s41598-018-20189-4

**Published:** 2018-02-02

**Authors:** Alison P. A. Menezes, Luciana C. Resende-Moreira, Renata S. O. Buzatti, Alison G. Nazareno, Monica Carlsen, Francisco P. Lobo, Evanguedes Kalapothakis, Maria Bernadete Lovato

**Affiliations:** 1 Departamento de Biologia Geral, Universidade Federal de Minas Gerais, CP 486, Belo Horizonte, MG 31270–901 Brazil; 2 Universidade de São Paulo, Instituto de Biociências, Departamento de Botânica, São Paulo, São Paulo, Brazil; 3 Smithsonian Institution, Botany Department, National Museum of Natural History, Washington, D. C., United States of America; 4 Present Address: Science and Conservation Division, Missouri Botanical Garden, St. Louis, Missouri United States of America

## Abstract

*Byrsonima* is the third largest genus (about 200 species) in the Malpighiaceae family, and one of the most common in Brazilian savannas. However, there is no molecular phylogeny available for the genus and taxonomic uncertainties at the generic and family level still remain. Herein, we sequenced the complete chloroplast genome of *B*. *coccolobifolia* and *B*. *crassifolia*, the first ones described for Malpighiaceae, and performed comparative analyses with sequences previously published for other families in the order Malpighiales. The chloroplast genomes assembled had a similar structure, gene content and organization, even when compared with species from other families. Chloroplast genomes ranged between 160,212 bp in *B*. *crassifolia* and 160,329 bp in *B*. *coccolobifolia*, both containing 115 genes (four ribosomal RNA genes, 28 tRNA genes and 83 protein-coding genes). We also identified sequences with high divergence that might be informative for phylogenetic inferences in the Malpighiales order, Malpighiaceae family and within the genus *Byrsonima*. The phylogenetic reconstruction of Malpighiales with these regions highlighted their utility for phylogenetic studies. The comparative analyses among species in Malpighiales provided insights into the chloroplast genome evolution in this order, including the presence/absence of three genes (*infA*, *rpl32* and *rps16*) and two pseudogenes (*ycf1* and *rps19*).

## Introduction

The chloroplast is an organelle that belongs to the family of plastids, playing an essential part in plant growth and development. Its main role is the photosynthesis, but it is also responsible for synthesis of amino acids, fatty acids, lipid components of their membranes and pigments, besides participating in the assimilation of nitrogen^1^. This organelle possesses its own genetic material, a circular and double-stranded DNA molecule, comprising about 120 genes (encoding ribosomal RNA, transfer RNA and proteins), and ranging in size between 107–218 kb^2^. Chloroplast genomes commonly present a highly conserved quadripartite structure formed by two inverted repeats (IRa and IRb), one large and another small single copy region (LSC and SSC, respectively)^3^. Nevertheless, some structural rearrangements may be observed, such as inversions, translocations, variation in copy number of tandem repeats and indels^4^. Chloroplast genome sequencing has contributed to solve phylogenetic and taxonomic problems in several groups^5–7^, to identify species by providing barcodes^8,9^ and to help in the conservation of endangered species^10^.

Malpighiales is a large order of Angiosperms^11^ and, partly because of its size, many of the phylogenetic relationships between its members are still not resolved^12^. The family Malpighiaceae Juss. is the third largest of the order^5^ and due to its high ecological and morphological diversity the family presents some taxonomic difficulties^13^. Morphological^13–15^ and molecular^16,17^ data support the monophyly of Malpighiaceae, although they are not sufficient to resolve relationships among groups within the family^17^. Davis and Anderson^17^ suggested the use of a large number of slow evolving genes to help solving phylogenetic relationships within the family. Such type of markers could be chloroplast genes, due its generally slow evolutionary rates. However, to date, no chloroplast genome of the family Malpighiaceae has been published.

*Byrsonima* Rich. ex Kunth (popularly known as “murici” in Brazil) is one of the largest genera within the family Malpighiaceae^18^, including about 200 species. Native to the American continent, the genus has 97 species occurring in Brazil^19^, seven of which are endangered^20^. Up to now there are only two studies addressing the taxonomy and phylogeny of the genus^18,21^. In 1897, Niendzu^22^ proposed to split the genus into two subgenera, based on stamen morphology. More recently, Elias^18^ proposed to characterize two subgenera according to their flower color: one group (*Byrsonima* subg. *Macrozeugma*) with flowers displaying five, white or pink petals and the other (*Byrsonima* subg. *Byrsonima*) with all the petals, or just the posterior ones, yellow. Representing the first subgenus mentioned above, with pink flowers, there is *Byrsonima coccolobifolia* Kunth, popularly known as “murici-rosa”. On the other hand, *Byrsonima crassifolia* (L.) Kunth, commonly called “murici-amarelo”, is a typical representative of the subgenus *Byrsonima*. These two species have economic importance due to the use of their wood and fruits by both the food industry and popular trade^23,24^. Both species are common in Brazilian savannas (cerrado), including the disjunct savanna areas in the Amazon, where they are among the most common tree species^25^. Outside Brazil, there are occurrence records of *B*. *coccolobifolia* in Bolivia, Venezuela and Guyana^26^. *Byrsonima crassifolia* has a broader distribution, and is found from Mexico to Paraguay^27^. Despite its ecological and economic importance there is no molecular study addressing the taxonomy and phylogeny of the genus *Byrsonima*. The complete chloroplast genome of two *Byrsonima* subgenera representatives may be used to detect regions of high sequence divergence that could help resolve taxonomic uncertainties in the genus and in the Malpighiaceae family in general.

In the present study, we sequenced and performed a comparative analysis of the complete chloroplast genome of two species of the genus *Byrsonima*, *B*. *coccolobifolia* and *B*. *crassifolia*. We assessed regions of high sequence divergence between the two *Byrsonima* species to provide markers for phylogenetic and genetic studies. Furthermore, we compared the chloroplast genomes of Malpighiaceae family with those available for other families belonging to the Malpighiales in order to increase the knowledge about chloroplast genome evolution in this order and provide markers for further phylogenetic studies.

## Results

### Genome content and organization of the chloroplast genome in *Byrsonima* species

Sequencing of genomic libraries generated about 5GB (20 million reads) and 7GB (32 million reads) of raw data for *B*. *coccolobifolia* and *B*. *crassifolia*, respectively. The data was used to assemble both chloroplast genomes with a high mean coverage, 1074X for *B*. *coccolobifolia* and 805X for *B*. *crassifolia*.

**Figure 1:**
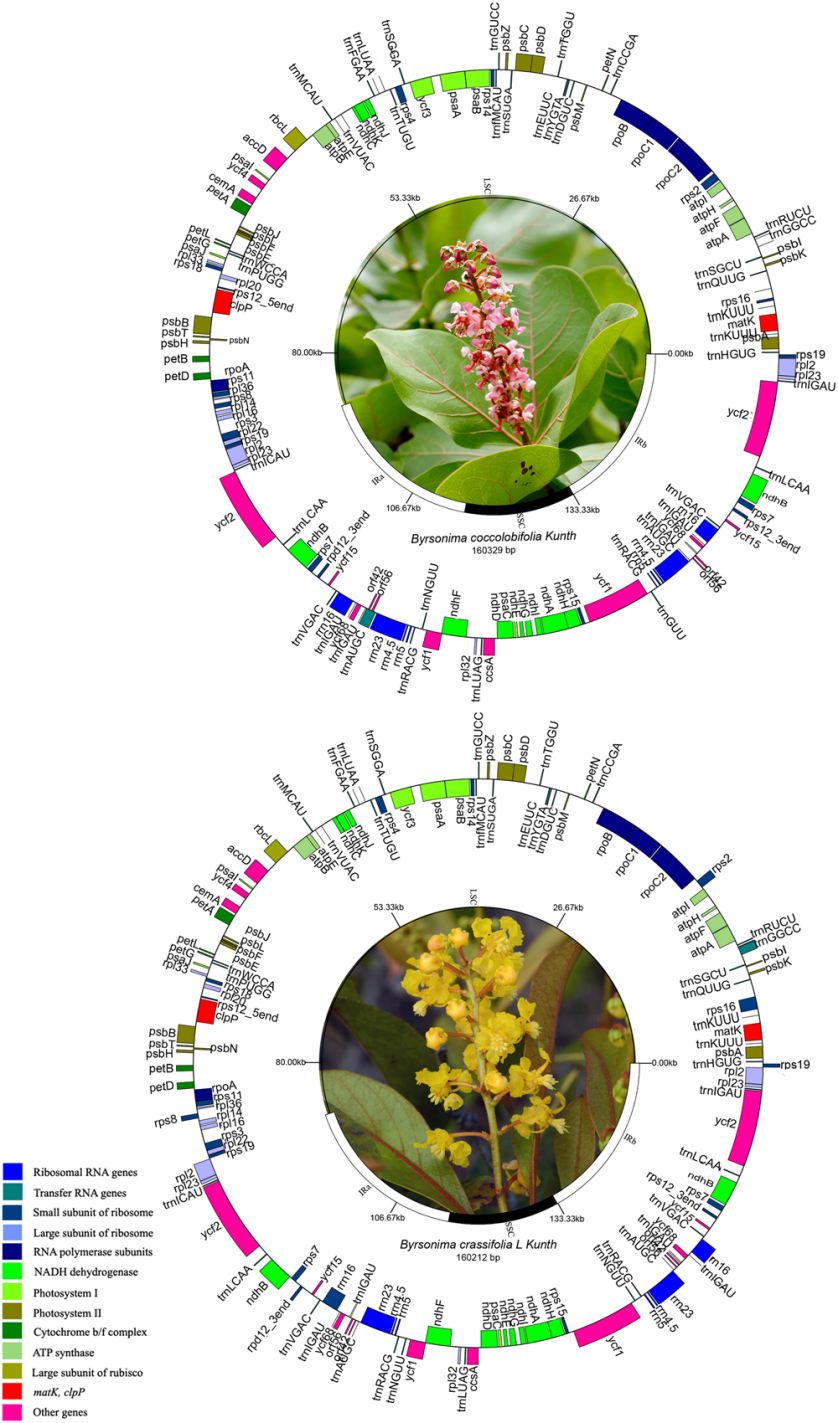
Chloroplast genome circular map of *Byrsonima coccolobifolia* Kunth and *B*. *crassifolia* (L.) Kunth (Malpighiaceae) with annotated genes. Genes inside the circle are transcribed clockwise, genes outside are transcribed counter-clockwise. Genes are color coded according to functional groups. Boundaries of the small (SSC) and large (LSC) single copy regions and inverted repeat (IRa and IRb) regions are noted in the inner circle for each species. Picture of *B*. *crassiflora* was taken by Dr. Daniel L. Nickrent (source: http://www.phytoimages.siu.edu). Picture of *B*. *coccolobifolia* was provided by Maurício Mercadante.

The chloroplast genomes of *B*. *crassifolia* and *B*. *coccolobifolia* exhibited similar structure and organization (Table 1, Fig. 1). The length of *B*. *coccolobifolia* chloroplast genome was 160,329 bp divided in four different regions, a pair of inverted repeated regions (IRa and IRb, 26,986 bp each) separated by two single copy regions, one large (LSC, 88,524 bp) and one small (SSC, 17,833 bp). The *B*. *crassifolia* chloroplast genome followed the same quadripartite structure, slightly shorter: IR was 26,975 bp each, LSC 88,448 bp and SSC 17,814 bp, for a total of 160,212 bp for the whole genome. The overall GC content was similar for the two species, 36.76% for *B*. *coccolobifolia and* 36.77% for *B*. *crassifolia*. Among the LSC, SSC and IR regions, the highest GC content was found in the IR regions (42.4% for both species). The GC content for the rRNA (55.42%) and tRNA (53.11%) genes was the highest among all coding regions, compatible to what has been observed in other studies^28–30^. This relatively higher GC content in rRNA and tRNA genes explains the higher GC content of IR regions, since they contain a great number of these genes.

**Table 1:** General information and comparison of chloroplast genomes of *Byrsonima coccolobifolia* and *B*. *crassifolia*.

Characteristics		*B*. *coccolobifolia*	*B*. *crassifolia*
Size (base pair; bp)		160329	160212
LSC length (bp)		88524	88448
SSC length (bp)		17833	17814
IR length (bp)		26986	26975
Number of genes		139	139
Protein-coding genes		94	94
tRNA genes		37	37
rRNA genes		8	8
Genes with intron(s)		18	18
GC content	Total (%)	36.76	36.77
	LSC (%)	34.53	34.52
	SSC (%)	30.66	30.76
	IR (%)	42.4	42.4
	CDS (%)	37.74	37.72
	rRNA (%)	55.42	55.42
	tRNA (%)	53.11	53.01
Coding protein genes (%bp)		50.2	50.2
Noncoding regions (%bp)		49.8	49.8

Both species displayed the same gene content and order (Table 2, Fig. 1), with 118 genes (four ribosomal RNA genes, 30 tRNA genes and 84 protein-coding genes), 21 of which are duplicated in the IR region. These species showed a bias towards using thymine (T) and adenine (A) in the third position of the codon; among the 20 amino acids, 11 of them used mostly codons ending with T and 7 used codons ending with A (Supplementary Table S1). This event is probably a result of an A + T rich genome, also observed in other chloroplast genomes studied^30–32^. The two genomes have 19 genes containing introns (Table 1), 15 with one intron and four with two or more introns. The *rpl32* gene (large ribosomal protein 32) contains three introns in *B*. *coccolobifolia* and four introns in *B*. *crassifolia*. In both species, the *rps12* gene (small ribosomal protein 12) is trans-spliced, that is, this gene has one intron, and the 5′ end exon is located in the LSC region while the second exon (3′ end exon) is located in the IRb (and therefore is duplicated in the IRa). We also detected 10 genes that partially overlap their sequences: *psbD*/*psbC*, *atpE/atpB*, *ycf1/ndhF*, *trnN-GUU/trnR-ACG* and *orf*4*2/trnA-UGC*. For both species the *ycf1* gene (5,745 bp) has its start in SSC region, but its sequence goes forward through SSC/IRa boundary, causing a duplication of the 3′ end portion of the *ycf1* gene in IRb and, therefore, producing a 1,389 bp *ycf1* pseudogene.

**Table 2:** Chloroplast genome gene content and functional classification in *Byrsonima coccolobifolia* Kunth and *B*. *crassifolia* (L.) Kunth.

Gene group	Gene name			
Ribosomal RNA genes	***rrn4***.***5***	***rrn5***	***rrn16***	***rrn23***
Transfer RNA genes	***trnA-TGC*** *	*trnC-CGA*	*trnD-GTC*	*trnE-TTC*
	*trnF-GAA*	*trnfM-CAT*	trnG-GCC*	*trnG-TCC*
	*trnH-GTG*	***trnI-CAT*** *	trnK-UUU*	***trnL-CAA***
	trnL-TAA*	*trnL-TAG*	*trnM-CAT*	***trnN-GTT***
	*trnP-GGG*	*trnP-TGG*	*trnQ-TTG*	***trnR-ACG***
	*trnR-TCT*	*trnS-GCT*	*trnS-GGA*	*trnS-TGA*
	*trnT-GGT*	*trnT-TGT*	***trnV-GAC***	trnV-TAC*
	*trnW-CCA*	*trnY-GTA*		
Small subunit of ribosome	*rps2*	*rps3*	*rps4*	***rps7***
	*rps8*	*rps11*	***rps12*** *	*rps14*
	*rps15*	rps16*	*rps18*	***rps19***
	***rps12_3end***			
Large subunit of ribosome	***rpl2*** *	*rpl14*	*rpl16*	*rpl20*
	*rpl22*	***rpl23***	rpl32*	*rpl33*
	*rpl36*			
RNA polymerase subunits	*rpoA*	*rpoB*	rpoC1*	*rpoC2*
NADH dehydrogenase	ndhA*	***ndhB*** *	*ndhC*	*ndhD*
	*ndhE*	*ndhF*	*ndhG*	*ndhH*
	*ndhI*	*ndhJ*	*ndhK*	
Photosystem I	*psaA*	*psaB*	*psaC*	*psaI*
	*psaJ*	ycf3*		
Photosystem II	*psbA*	*psbB*	*psbC*	*psbD*
	*psbE*	*psbF*	*psbH*	*psbI*
	*psbJ*	*psbK*	*psbL*	*psbM*
	*psbN*	*psbT*	*psbZ*	
Cytochrome b/f complex	*petA*	*petB*	*petD*	*petG*
	*petL*	*petN*		
ATP synthase	*atpA*	*atpB*	*atpE*	atpF*
	*atpH*	*atpI*		
Large subunit of rubisco	*rbcL*			
Maturase	*matK*			
Protease	clpP*			
Envelope membrane protein	*cemA*			
Subunit of acetyl-CoA-carboxylase	*accD*			
c-type cytochrome synthesis	*ccsA*			
Component of TIC complex	***ycf1***	*ycf1* ^*Ψ*^		
Hypothetical chloroplast reading frames	***ycf2***			
ORFs	***orf42***	***orf56*** *	*ycf 4*	***ycf15*** *
	***ycf68*** *			

*Genes containing introns; ^Ψ^Pseudogene; genes in bold are located within the IR and therefore are duplicated.

### Comparative analysis of chloroplast genomes within Malpighiales

**Figure 2:**
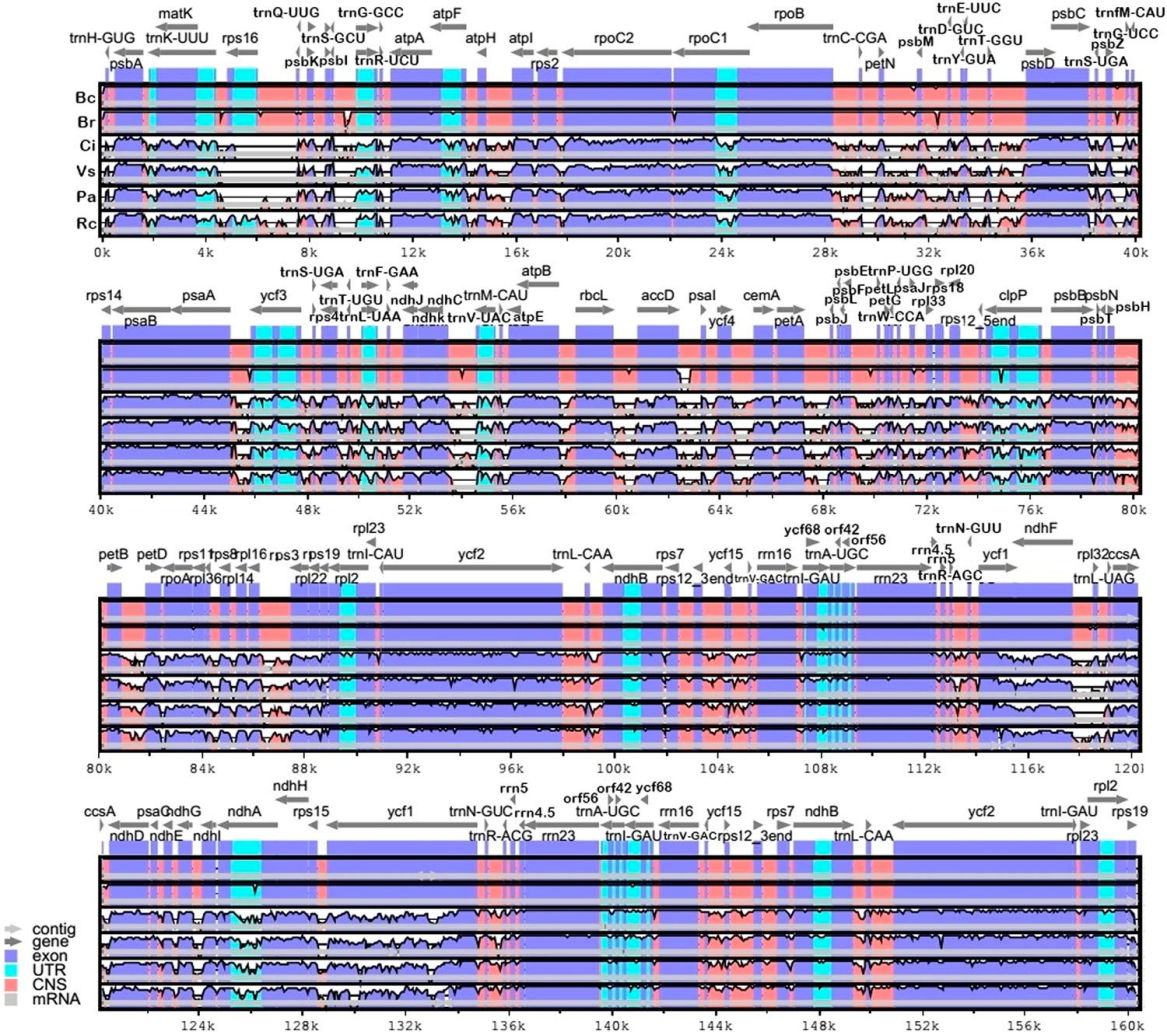
Comparisons of percentage identity of chloroplast genomes for six species belonging to five different families within the order Malpighiales. Bc: *Byrsonima coccolobifolia*; Br: *Byrsonima crassifolia* (Malpighiaceae); Ci: *Chrysobalanus icaco* (Chrysobalanaceae); Vs: *Viola seoulensis* (Violaceae); Pa: *Populus alba* (Salicaceae), Rc: *Ricinus communis* (Euphorbiaceae). The percentage of identity is shown in the vertical axis, ranging from 50% to 100%, while the horizontal axis shows the position within the chloroplast genome. Each arrow displays the annotated genes and direction of their transcription in the reference genome (*Byrsonima coccolobifolia*). Genome regions are color coded as exon, untranslated region (UTR), conserved noncoding sequences (CNS) and mRNA.

**Figure 3:**
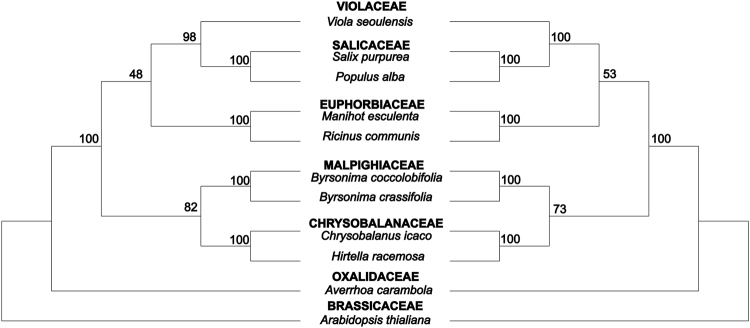
Maximum likelihood trees for the order Malpighiales inferred from complete chloroplast genomes of nine species of the order (using all putative 1–1 orthologs - right) and from five highly variable coding sequences identified in this study (*accD*, *matK*, *rpoA*, *ycf2* and *rps7* - left). Bootstrap values are indicated above branches.

The analysis performed on the mVista software^33^ showed the level of similarity for the whole sequence of the chloroplast genome of the nine Malpighiales species analyzed (Supplementary Fig. S1). We observed highly conserved sequences within the families, thus to facilitate visualization we only include one member of each family and the two *Byrsonima* species in Fig. 2. Overall, the comparative genomic analyses showed low sequence divergence between the two *Byrsonima* species. The highest levels of divergence were found in intergenic regions, namely *psbK-psbI*, *trnS-trnR*, *rpoC1-rpoC2*, *trnY-trnE*, *accD-psaI*, *psaJ-rpl33* and *clpP* intronII. Apart from these regions, when comparing the nine species from different families, we observed some coding sequences with low similarity levels (below 70%): *accD*, *matK*, *rpoA*, *ycf2*, *ycf1* and *rps7*. Phylogenetic relationships within the order, reconstructed using these coding regions showed a concordant topology and boostrap values similar to the results obtained with complete chloroplasts, derived from all 1–1 orthologs (62 groups) (Fig. 3).

Evolutionary rates varied widely among genes across the nine Malpighiales species analyzed (Supplementary Table S2). In general, the Ka/Ks values were lower than 0.5 for almost all genes (ca. 90%). Six genes related to photosynthesis (*psbD*, *psbE*, *psbF*, *psbL*, *psbN* and *psbT*) presented the lowest evolutionary rates (Ka/Ks = 0.0002 to 0.07), exhibiting a uniform rate across most of the species evaluated. Nineteen genes returned Ka/Ks rates higher than 0.5 and lower than 1 in at least one of the species. The genes *rps14*, *psaI*, *cemA*, *rpl*23, *ycf2*, *ycf15*, *ycf68* and *ycf1* showed Ka/Ks rate higher than 0.5 and lower than 1 for three or more species. The genes *matK*, *clpP*, *infA* and *ccsA* showed Ka/Ks values higher than 1 for one species and other five genes (*atpE*, *ycf15*, *ycf68*, *orf42* and *ycf1*) presented these high rates for at least two species. The two *Byrsonima* species showed similar substitution rates and Ka/Ks ratio for most genes (ca. 77%), except for 25 genes that showed differences in Ka/Ks ratio higher than 5%. Fifteen of these genes (*rps4*, *ndhJ*, *rbcL*, *accD*, *cemA*, *clpP*, *psbJ*, *petD*, *rps11*, *rpl22*, *rpl2*, *ycf68*, *orf56*, *ccsA* and *ndhI*) were evolving faster in *B*. *coccolobifolia* than in *B*. *crassifolia*, on the other hand, ten genes (*rps16*, *rpoC1*, *ndhK*, *atpE*, *rpoA*, *rps3*, *rps7*, *ndhF*, *rpl32* and *ndhA*) were evolving faster in *B*. *crassifolia*.

**Figure 4:**
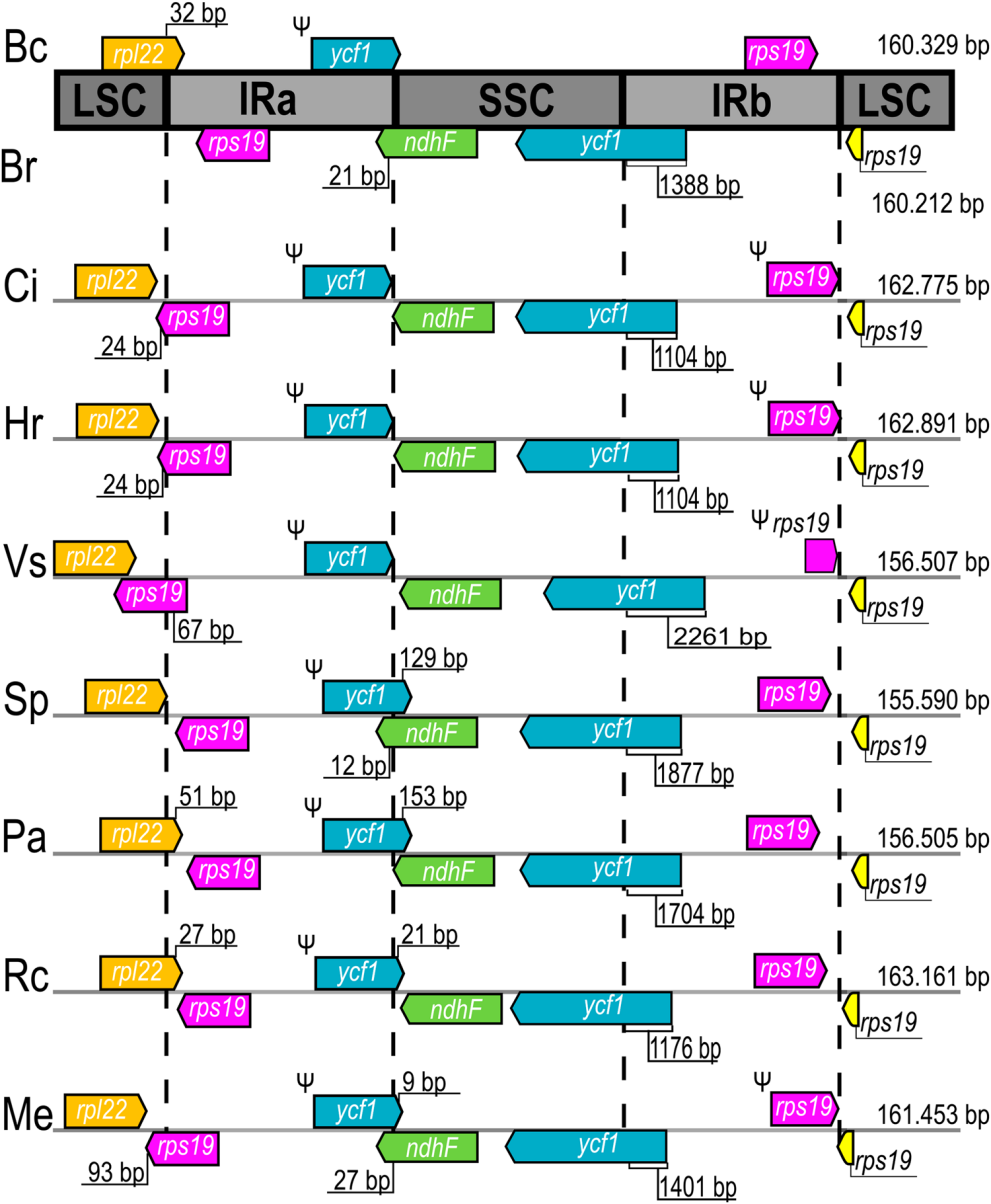
Details of boundary positions between inverted repeat regions (IR) and large and small single copy regions (LSC and SSC) among nine chloroplast genomes within the order Malpighiales. Bc: *Byrsonima coccolobifolia*; Br: *B*. *crassifolia* (Malpighiaceae); Ci: *Chrysobalanus icaco*; Hr: *Hirtela racemosa* (Chrysobalanaceae); Vs: *Viola seoulensis* (Violaceae); Sp: *Salix purpurea*, Pa: *Populus alba* (Salicaceae), Rc: *Ricinus communis*, Me: *Manihot esculenta* (Euphorbiaceae). Both *Byrsonima* species sequences are represented together at the top of the figure given that there are no differences between their boundaries. The direction of arrows shows the direction of transcription (right is forward and left is reverse). Ψ indicates a pseudogene. Length of arrows is illustrative. Number of base pairs (bp) indicates distance from the boundary to the end of the gene. Complete chloroplast genome sizes are noted on the right-hand side of the panel.

Figure 4 shows a comparison between boundary regions of the chloroplast genome of species in the order Malpighiales. The position of the SSC/IRb junction in all compared species is found within the *ycf1* gene, therefore creating a pseudogene of the 5′ end of this gene (*ycf1*^*Ψ*^) in the IRa region. The *ycf1*^*Ψ*^ size varies from 1,104 bp (in *Chrysobalanus icaco* L. and *Hirtella racemosa* Lam.) to 2,261 bp (*Viola seoulensis* Nakai). In both *Byrsonima* species the *ycf1*^*Ψ*^ size was the same length, 1,388 bp. Regarding the LSC/IRa borders in *B*. *coccolobifolia* and *B*. *crassifolia*, they are located in the 3′ end of *rpl22* gene, duplicating 32 nucleotides of this gene in the IRb. *Populus alba* L. and *Ricinus communis* L. showed the same pattern as the *Byrsonima* species. In *C*. *icaco*, *H*. *racemosa* and *Manihot esculenta* Crantz the location of LSC/IRa junction is in the 3′ end of the *rps19* gene, thus creating an *rps19* pseudogene in the IRb region. *Salix purpurea* L. presents the LSC/IRa boundary in the intergenic space between *rpl22* and *rps19*. In *V*. *seoulensis* the limit between LSC and IR regions is located in the 5′ end of the *rps19* gene, turning the gene copy in the IRb region into a pseudogene of 67 bp.

### Repeated sequences analysis of *Byrsonima* species

The IMEx software^34^ found 427 small single repeats (SSR) in the *B*. *coccolobifolia* chloroplast genome and 414 in *B*. *crassifolia* (Supplementary Table S3). Most of the SSR discovered were mononucleotide repetitions (ca. 79%), varying from seven to 16 nucleotides long. About 57% of the SSR were mononucleotides sequences containing repetitions of adenine (A) or thymine (T). Repeats of di- and tri-nucleotides were also abundant, representing together 20% of the SSR found for both species. For dinucleotide SSR, the number of repeats ranged from four to seven, but for tri-, tetra- and penta-nucleotide SSRs, they had mostly three motif repetitions, except for two sequences with four repeats (Supplementary Table S3). The REPuter^35^ screening discovered three categories of dispersed repeats: forward (F), palindrome (P) and reverse (R) (Table 3). In the *B*. *coccolobifolia* chloroplast genome we found 15 repeats (F = 6; P = 8; R = 1) and 19 in *B*. *crassifolia* (F = 9; P = 9; R = 1), with motif length ranging from 30 bp to 57 bp. Most of the repeated sequences were located in the *ycf2* gene (18 for each species) and intergenic spacers (IGS) (10 and 18, for *B*. *coccolobifolia* and *B*. *crassifolia*, respectively).

**Table 3:** Distribution of repeated sequences in the chloroplast genome of *Byrsonima coccolobifolia* and *B*. *crassifolia*.

Type	Location	Region	Repeated sequence	Size (bp)
F	ycf2	IRa	ATATCGTCACTATCATCAATATCGTCACTATCATCAATATCGTCACTATCATCAATA	57
P	ycf2	IRa/IRb	TATTGATGATAGTGACGATATTGATGATAGTGACGATATTGATGATAGTGACGATAT	57
P	ycf2	IRa/IRb	TATTGATGATAGTGACGATATTGATGATAGTGACGATATTGATGATAGTGACGATAT	57
F	ycf2	IRb	ATATCGTCACTATCATCAATATCGTCACTATCATCAATATCGTCACTATCATCAATA	57
P	trnQ-rps16	LSC	AGAGATCTAATCCCATTGATTGAATTCAATCAATGGGATTAGATCTCT	48
F	trnS-trnQ*	LSC	TATACTATTAGATACTACTATATACTATTAGTATACTATTAGATACTA	48
P	petN-trnT*	LSC	AGATAGTATGGTAGAAAGAAATATATATATTTCTTTCTACCATACTAT	48
P	petA-petL	LSC	CTTTTCGATTTTATACGTATAAATTTATACGTATAAAATCGAAAAG	46
F	ycf2	IRa	ATATCGTCACTATCATCAATATCGTCACTATCATCAATA	39
P	ycf2	IRa/IRb	TATTGATGATAGTGACGATATTGATGATAGTGACGATAT	39
P	ycf2	IRa/IRb	TATTGATGATAGTGACGATATTGATGATAGTGACGATAT	39
F	ycf2	IRb	ATATCGTCACTATCATCAATATCGTCACTATCATCAATA	39
R	rbcL-accD	LSC	AGAATTAAGAGAATTAAAATTAAGAGAATTAAGA	34
F	psaB and psaA	LSC	ACCGATATTGCACACCATCATTTAGCTATTGCA	33
P	petN-psbM	LSC	TTTAATTTAAATTGAATTCAATTTAAATTAAA	32
P	trnR-trnS and ycf2	LSC/IRa	ATATATGTTTGGAATAGATTCCATTTTGAGA	31
F	trnR-trnS and ycf2	LSC/IRa	TCTCAAAATGGAATCTATTCCAAACATATAT	31
F	psbK-psbI*	LSC	ATACTATTAGATACTACTATATACTATTAG	30
F	psbK-psbI*	LSC	ATACTATTAGATACTACTATATACTATTAG	30

*Repeats that appear only in *B*. *crassifolia*. Types of repeats are F (forward), P (palindrome) and R (reverse).

### Highly divergent regions between *Byrsonima* species

The level of divergence between the two *Byrsonima* species was variable depending on the region of the chloroplast compared (Supplementary Fig. S2), with nucleotide diversity (π) ranging from 0.000345 (*rpoB* gene) to 0.065574 (*atpA-atpF* intergenic spacer). The IGS showed higher average π (0.002664) than the protein coding (0.000623), intronic (0.000895) and tRNA regions (which proved to be very conserved, π = 0). Among the 20 regions with the highest values of π (all > 0.005), 18 were IGS and only two were protein coding genes (Table 4). Some regions exhibited neighboring sequences with high π values (Supplementary Fig. S2). Thus, we calculated the divergence values for the combined tandem sequences. Among these tandems, one region of 625 bp between the genes *rpoA* and *rpl36* exhibited a high π value (0.011475 – Table 4). The gene *ycf1* showed no divergence between the two *Byrsonima* species (π = 0), whereas its *ycf1*^*Ψ*^ pseudogene, located in the IRb, had a π of 0.002747, higher than the average for IGS.

**Table 4:** Twenty most divergent regions of chloroplast genome based on a comparison between *Byrsonima coccolobifolia* Kunth and *B*. *crassifolia* (L.) Kunth.

Region	Nucleotide diversity (π)	Total number of mutations (η)	Region length (bp)
*atpA-atpF*	0.065574	4	61
*ccsA-ndhD*	0.040000	10	250
*rpoA-rps11*	0.029851	2	80
*psbT-psbN*	0.015385	1	65
*trnH-GUG-psbA*	0.014337	4	279
*psbI-trnS-GCU*	0.011765	1	85
*trnG-UCC-trnfM-CAU*	0.011765	2	172
*rpoA-rps11-rpl36*	0.011475	7	625
*psbZ-trnG-UCC*	0.011050	6	712
*rps11*	0.009639	4	417
*psaI-ycf4*	0.008869	4	453
*rpl32-trnL-UAG*	0.007874	3	381
*rps11-rpl36*	0.007813	1	128
*rpl14-rpl16 exon II*	0.007246	1	139
*trnK-UUU-rps16*	0.006289	3	518
*psaJ-rpl33*	0.005859	3	555
*petD-rpoA*	0.005682	1	176
*matK-trnK-UUU*	0.005587	4	716
*rpl32*	0.005556	1	184
*rps16-trnQ-UUG*	0.005178	8	1,575

## Discussion

In this study, the whole chloroplast genomes of *Byrsonima coccolobifolia* and *B*. *crassifolia* were sequenced and analyzed. The comparative analysis of these genomes and other species of the order Malpighiales has brought insights about chloroplast genome evolution in this order. Moreover, this study identified sequences suitable for use in future evolutionary studies in the order Malpighiales, in the family Malpighiaceae and in the genus *Byrsonima*, in order to clarify phylogenetic relationships and resolve taxonomic uncertainties.

Although gene content and organization were generally similar in the species analyzed within the order Malphighiales, some striking differences were found among them. One remarkable variation among the species analyzed is the presence or absence of three protein coding genes. The *rps16* and *rpl32* genes were absent in the single Violaceae species analyzed (*V*. *seoulensis*) and also in the Salicaceae family (*P*. *alba* and *S*. *purpurea*). The gene *infA* was lacking in both species of Malpighiaceae, *B*. *coccolobifolia*, *B*. *crassifolia*, in *V*. *seoulensis* and in one of the two species of Euphorbiaceae, *M*. *esculenta*. Thus, the evolutionary change leading the absence/presence of *infA* gene in the chloroplast genome even within a family appears to have occurred several times within the order Malpighiales. The absence of some genes, including these three particular genes, has been described in other plant species^36–40^. Some studies have shown that *infA*^41^, *rpl32*^38^ and *rps16*^42^ genes that were missing in the chloroplast genome of certain species have been transferred to the nuclear genome. Further investigation will be needed to check if the three genes lacking in the chloroplast genome of these Malpighiales species analyzed were transferred to another genome compartment or were completely lost.

Another important characteristic of the chloroplast genome that is useful for evolutionary studies is the location of the boundaries among the four chloroplast regions. Evaluating their contraction and expansion can shed some light on the evolution of some taxa^32^. From our results we noticed that the length variation in the IR regions created some pseudogenes, like the *ycf1*^*Ψ*^ or *rps19*^*Ψ*^. The *ycf1*^*Ψ*^ pseudogene is present in all studied species, whereas the *rps19*^*Ψ*^ pseudogene is only present in *C*. *icaco*, *H*. *racemosa* (Chrysobalanaceae), *V*. *seoulensis* (Violaceae) and *M*. *esculenta* (Euphorbiaceae); in the other Malpighiales species the *rps19* gene is fully duplicated. Thus, the contraction/expansion of IR regions, creating pseudogenes, has occurred more than once in the order Malpighiales.

Even though, as expected, sequence divergence among families was higher than within a family, in general, the chloroplast genomes within Malpighiales are still conserved, as observed in other flowering plants^2^. High levels of divergence among families were found for the *accD*, *matK*, *rpoA*, *ycf2*, *ycf1* and *rps7* genes. Most of these sequences have already been used for phylogenetic studies^43–46^, and our analyses showed that these regions (excluding *ycf1*) were in fact very informative for inferring phylogenetic relationships within the order, with results comparable to those obtained from complete chloroplast genomes. Moreover, these topologies were concordant with the most complete phylogenetic study performed for the order so far^5^. These results highlight the utility of the highly divergent regions identified herein for phylogenetic inference in the Malpighiales order.

The slow evolutionary rates and the low Ka/Ks ratio detected in the Malpighiales species analyzed are expected for chloroplast genomes in general^47^. The genes with the lowest evolutionary rates were photosynthesis genes (*psbD*, *psbE*, *psbF*, *psbL*, *psbN* and *psbT*), an evolutionary pattern common in photosynthetic plants^48^. Among the genes with highest evolutionary rates *ycf1*, *ycf15* and *ycf68* do not have a known function and its high Ka/Ks ratio may show that they play a non-essential role in plant cells. These results, together with the differences found between the two *Byrsonima* species in Ka/Ks ratios for 25 genes, are evidence that evolutionary rates in the chloroplast genome in Malpighiales vary strongly among genes and lineages.

Repetitive sequences have been reported in the chloroplast genome of many plant lineages^49,50^. These types of markers are used for a wide range of evolutionary and population genetic studies^51,52^. *Byrsonima coccolobifolia* and *B*. *crassifolia* showed the same motifs of SSR markers, but in general the *B*. *coccolobifolia* chloroplast genome presented more SSR loci than *B*. *crassifolia*. In terms of dispersed repeats, both species shared most of the repeated sequences, but three repeats were found only in *B*. *crassifolia*. Interestingly, dispersed repeats were found mainly in protein coding sequences, and 18 (of the 30 repeats in *B*. *coccolobifolia* and 36 in *B*. *crassifolia*) were contained in the *ycf2* genes, whereas other two were found in the *psaA* and *psaB* genes. This result does not follow the tendency of organelar genomes, since most repeated sequences in chloroplast genomes are located in intergenic sequences^53–55^. However, a greater amount of dispersed repeats was also found in coding sequences in five species of the genus *Epimedium* L. (Berberidaceae^7^).

Based on the comparison of nucleotide diversity among regions between the two *Byrsonima* species analyzed, we suggest a set of 20 regions with high divergence, most of them intergenic sequences, to be used as a starting point for investigating potential markers for phylogenetic and phylogeographic studies in the genus *Byrsonima*. Until now, there has been no phylogenetic study of this genus, and taxonomic uncertainties still remain^18^. To look for polymorphic sequences in the chloroplast of some species is usually very time-consuming when no previous chloroplast genome information is available. In fact, a recent study by our group^56^ observed only three polymorphic regions after testing 15 of the most commonly used chloroplast regions for a phylogeographic study in *B*. *coccolobifolia* populations. The lack of available sequences for these regions hindered us from testing their utility in a phylogenetic context, but we expect that the highly divergent sequences identified here by comparison of *B*. *cocolobifolia* and *B*. *crassifolia* chloroplast genomes will offer new tools for genetic and evolutionary studies in species of this genus and of the Malpighiaceae family.

## Material and Methods

### Sample material and sequencing

Samples used in the study were collected in Amazonian savanna enclaves: *Byrsonima coccolobifolia* (voucher BHCB 169523) from Boa Vista (60°49′45′′W, 2°39′40′′N) and *B*. *crassifolia* (voucher BHBC 169445) from Alto Alegre (61°09′04′′W, 3°09′45′′N). Voucher specimens were deposited in BHCB herbarium (Herbarium of Departamento de Botânica, Universidade Federal de Minas Gerais). Genomic DNA was extracted from silica-dried leaves, using Novaes *et al*.^57^ protocol. DNA quality was assessed in a spectrophotometer Nanodrop 2000 (Thermo Scientific) and integrity was evaluated using a 0.8% agarose gel. In addition, DNA was quantified through fluorometry using Qubit 2.0. (Life Technologies). DNA samples from each species were used to prepare two separate libraries with Nextera kit (Illumina Inc., San Diego, CA), following manufacturer’s protocol. Different barcodes were used to identify DNA fragments derived from each species. To guarantee the intended fragments size, aliquots of each library were ran in 1% agarose gel and quantified by quantitative PCR, using a Library Quantification Kit – Illumina/Universal (Kapa Biosystems Inc., Wilmington, MA). Short fragments of approximately 600 bp from both libraries were combined and submitted for paired-end sequencing using a single lane on a MiSeq sequencer (Illumina Inc.).

### Genome assembly and annotation

Raw sequences were submitted to the Sequence Read Archive (SRA accession number SRP109225). Pair-end Illumina raw reads where cleaned from adaptors and barcodes and then quality filtered using Trimmomatic^58^. Reads were trimmed from both ends, and individual bases with Phred quality score < 20 were removed, as well as more than three consecutive uncalled bases. Entire reads with a median quality score lower than 21 or less than 40 bp in length after trimming were discarded. After quality filter, reads were mapped to the chloroplast genome of the closest species with a chloroplast genome available (*Chrysobalanus icaco* L. – Chrysobalanaceae Juss.), using Bowtie2 v.2.2.6^59^ in order to exclude reads of nuclear and mitochondrial origins. All putative chloroplast reads mapped to the *Chrysobalanus* reference above were then used for *de novo* assembly to reconstruct *Byrsonima* chloroplast genomes using SPAdes 3.6.1^60^ with iterative K-mer sizes of 55, 87 and 121. *De novo* assembled chloroplast contigs were concatenated into larger contigs using Sequencher 5.3.2 (Gene Codes Inc., Ann Arbor, MI, USA) based on at least 20 bp overlap and 98% similarity. A “genome walking” technique using the Unix “grep” function was able to find any remaining reads that could fill any gaps between contigs that did not assemble in the initial set of analyses. Read coverage analysis was then conducted to determine the inverted repeat (IR) region boundaries and any misassembled contigs using Jellyfish v.2.2.3^61^ and pipeline developed by M. McKain (https://github.com/mrmckain/Chloroplast-Genome-Assembly/tree/master/Coverage_Analysis).

Automatic annotation of *B*. *coccolobifolia* and *B*. *crassifolia* chloroplast genomes were generated by CpGAVAS^62^ and a circular representation of both sequences was drawn using the online tool GenomeVX^63^. The draft annotations given by CpGAVAS were then manually corrected using the Artemis software^64^ and other plastid genomes for comparison. The complete chloroplast genomes of *B*. *coccolobifolia* and *B*. *crassifolia* were automatically annotated and aligned in Verdant^65^. Differences between results from CpGAVAS and Verdant were manually confirmed and investigated in GenBank when necessary. Open reading frames identified by these softwares were reported when sequences followed two criteria: (1) have been described previously in other chloroplast genomes^32,66^, (2) were homologous to known genes (using the BLAST tool from GenBank). The complete chloroplast genome sequence and final annotations for both species were submitted to GenBank under the following accession numbers: MF359247 (*B*. *coccolobifolia*) and MF359248 (*B*. *crassifolia*).

### Comparative analyses and evaluating regions of high divergence

Aiming to perform a comparative genomic analysis within the order Malpighiales, we chose two species of each family in the order with chloroplast genomes available on NCBI database: Euphorbiaceae, Chrysobalanaceae, Salicaceae and Violaceae (which had only one genome currently published – supplementary Table S4). Then, we used the software mVISTA in Shuffle-LAGAN mode^33^, with default parameters for other options, to compare the chloroplast genomes from the five different plant families, using the newly sequenced *B*. *coccolobifolia* annotated genome as a reference. In order to detect expansion or contraction of the IR regions boundaries between the four main parts of the annotated chloroplast genomes (LSC, IRa, SSC and IRb) were visually inspected among the nine species in the order Malpighiales using the Artemis software^64^.

The protein coding regions of these same nine chloroplast genomes were used to evaluate evolutionary rate variation within Malpighiales. For that, we calculated the rates of non-synonymous (Ka) and synonymous substitutions (Ks), as well as their ratio (Ka/Ks) using Model Averaging in the KaKs_Calculator^67^. In this instance, the Malpighiales plant species *Passiflora edulis* Sims (NC_034285.1) was used as a reference, and alignments of the protein-coding sequences (without stop codons) from the nine species were performed using the MUSCLE^68^ program in Mega 7^69^.

Further comparisons between *Byrsonima* species were performed with the repetitive elements found in their chloroplast sequences. To analyze the presence of perfect microsatellites we used the Imperfect Microsatellite Extractor (IMEx) interface^34^, with minimum thresholds of seven for mononucleotide repeats, four for dinucleotide repeats and three for tri-, tetra-, penta-, and hexanucleotide repeats. REPuter software^35^ was used to detect tandem repeats. We set the parameters to localize forward, reverse, complementary and palindromic sequences with a minimum distance of 30 bp and 90% minimum identity.

In order to identify regions of high genetic divergence between *Byrsonima* species that could potentially be informative for future phylogenetic studies in the genus, we calculated the genetic divergence between *B*. *coccolobifolia* and *B*. *crassifolia* across the entire chloroplast genome. Genetic divergence was calculated using nucleotide diversity (π) and total number of mutations (η) for coding genes, intron sequences and intergenic spacers (IGS) aligned with Verdant, using DnaSP 5.0^70^.

### Phylogenetic analysis

Phylogenetic relationships within the Malpighiales order were reconstructed using the complete set of species sampled in our comparative analysis (seven species available in NCBI plus the two *Byrsonima* described in our study) and two species of different orders as outgoup, *Averrhoa carambola* and *Arabidopisis thaliana* (KU569488.1 and NC000932.1, respectively). In order to evaluate the usefulness of the highly variable chloroplast regions identified within Malpighiales by mVista (*accD*, *matK*, *rpoA*, *ycf2*, *ycf1* and *rps7*), we compared phylogenies inferred from two matrices: one using five of these highly variable sequences and other using putative 1–1 orthologs genes within Malpighiales order. Because the highly variable sequence *ycf1* showed some inversions that hindered the alignment, we excluded this region of the phylogenetic analysis.

The highly variable sequences were extracted separately for each species, aligned using MUSCLE^68^ and concatenated to generate a matrix for the first input file. To create the 1–1 orthologs genes file, we extracted coding sequences from complete chloroplast genomes of 11 species and translated them using *in house* Perl scripts (available from the authors upon request). The protein sequences were used as input to OrthoMCL2 to predict homology relationships^71^. The groups of homologs that are present in one copy in all predicted chloroplast proteomes were considered as putative 1–1 orthologs (62 groups) and were individually aligned using MUSCLE^68^. We used the aligned protein sequences for each group to generate codon alignments using PAL2NAL^72^. Finally, we took the aligned codon sequences for each genome and concatenate them to generate a gene matrix that was used to create the second input file. Both alignment were verified and edited manually. The program PartitionFinder^73^ was used to identify the best-fit partitioning schemes and suitable evolution model for phylogeny estimation of each matrix. Finally, the best trees were inferred from Maximum likelihood (ML) analyses, performed with RAxML 8.3.2^74^ in CIPRES Science Gateway^75^, using GTR + G model and 1000 rapid bootstrap replications for each matrix.

### Data availability

The complete chloroplast sequences generated and analysed during the current study are available in GenBank, https://www.ncbi.nlm.nih.gov/genbank/ (accession numbers are described in the text).

## Electronic supplementary material

Supplementary information
