# Supplementary material for: Chloroplast genomes of Byrsonima species (Malpighiaceae): comparative analysis and screening of high divergence sequences
Source: Sci Rep. 2018 Feb 2;8:2210. doi: 10.1038/s41598-018-20189-4 (PMC5797077; doi:10.1038/s41598-018-20189-4)
Supplement: Supplementary file 1 — Supplementary information [file 41598_2018_20189_MOESM1_ESM.pdf]

Suplementary information

Title: Chloroplast genomes of *Byrsonima* species (Malpighiaceae): comparative analysis and screening of high divergence sequences

Alison P. A. Menezes[1,*], Luciana C. Resende-Moreira[1,*], Renata S. O. Buzatti[1,*], Alison G. Nazareno[2], Monica Carlsen[3,4], Francisco P. Lobo[1], Evanguedes Kalapothakis[1] & Maria Bernadete Lovato[1]

[1]Departamento de Biologia Geral, Universidade Federal de Minas Gerais, CP 486, Belo Horizonte, MG 31270-901, Brazil

[2]Universidade de São Paulo, Instituto de Biociências, Departamento de Botânica, São Paulo, São Paulo, Brazil

[3]Smithsonian Institution, Botany Department, National Museum of Natural History, Washington, D.C., United States of America

[4]Current address: Science and Conservation Division, Missouri Botanical Garden, St. Louis, Missouri, United States of America

*These authors contributed equally to this work.

Correspondence should be addressed to M.B.L. (email: lovatomb@icb.ufmg.br).

Suplementary figures

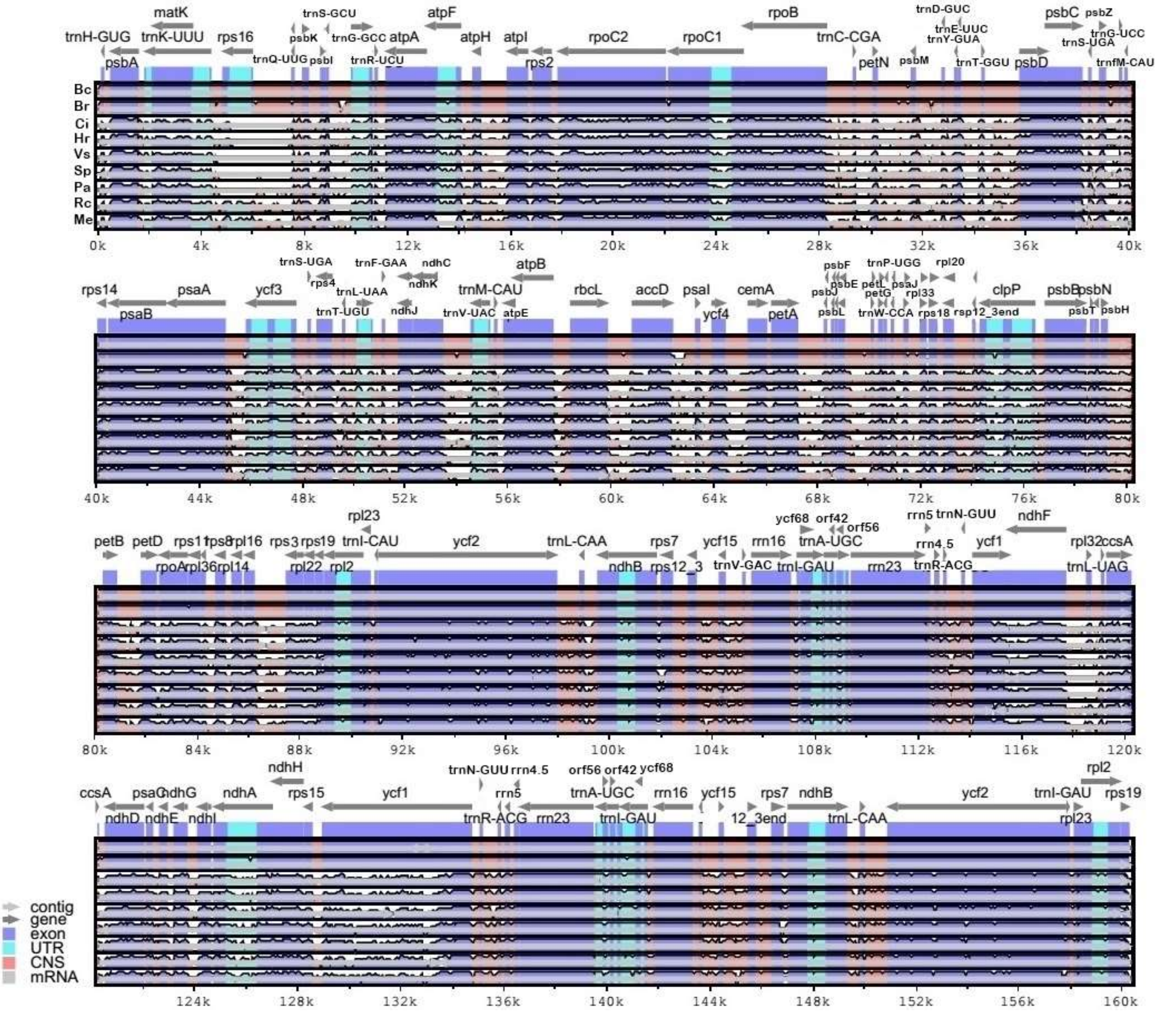

Figure S1 - Comparisons of percentage identity of chloroplast genomes for nine species belonging to five different families within Malpighiales order. Bc: *Byrsonima coccolobifolia* and Br: *B. crassifolia* (Malpighiaceae); Ci: *Chrysobalanus icaco* and Hr: *Hirtela racemosa* (Chrysobalanaceae); Vs: *Viola seoulensis* (Violaceae); Sp: *Salix purpurea* and Pa: *Populus alba* (Salicaceae); Rc: *Ricinus communis* and Me: *Manihot esculenta* (Euphorbiaceae). The percentage of identity is shown in the vertical axis, ranging from 50% to 100%, while the horizontal axis shows the position within the chloroplast genome. Each arrow displays the annotated genes and direction of their transcription in the reference genome (Byrsonima coccolobifolia). Genome regions are

color coded as exon, untranslated region (UTR), conserved noncoding sequences (CNS) and mRNA.

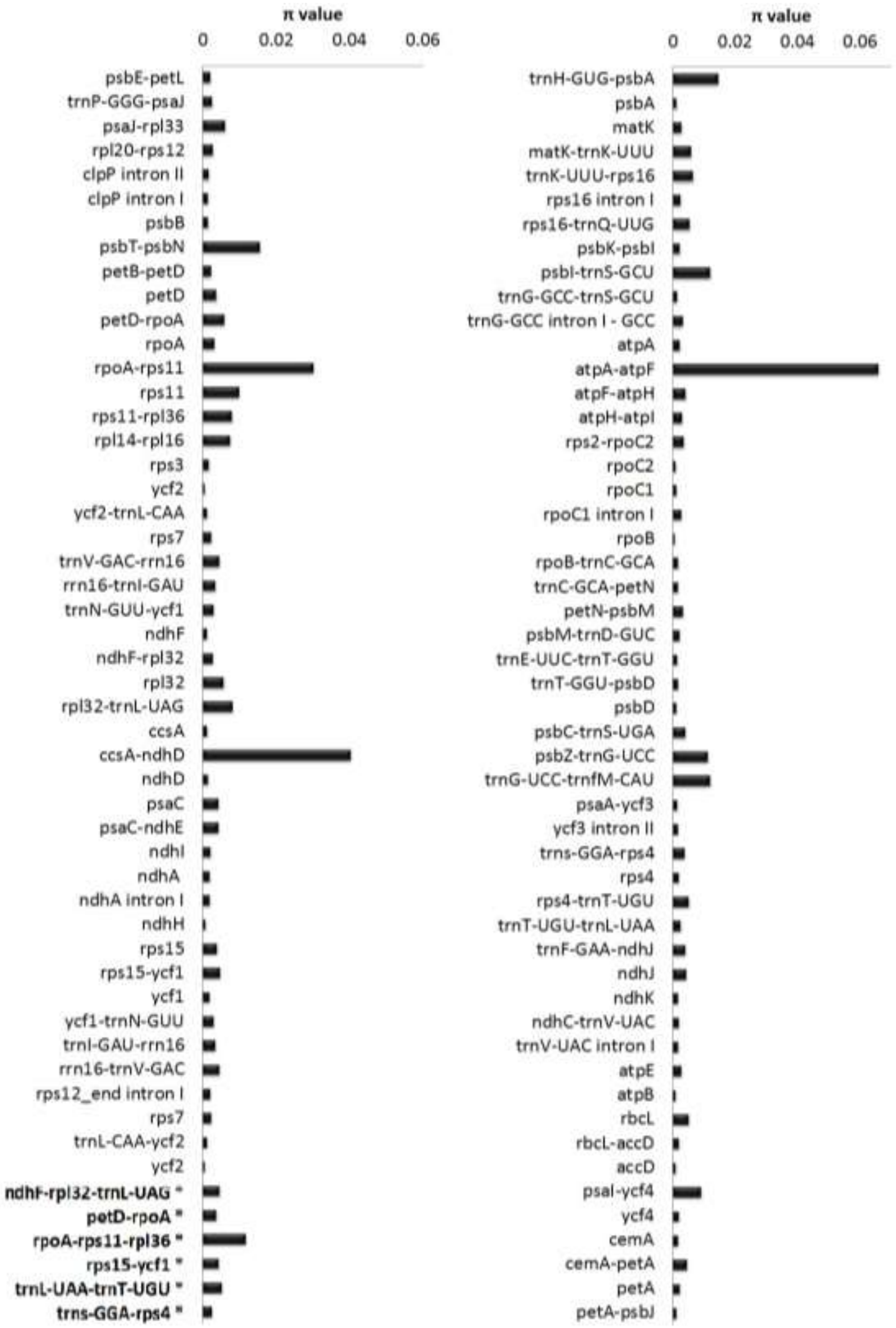

Figure S2 - Nucleotide divergence (π) between *Byrsonima coccolobifolia* and *B. crassifolia*. Sequences with π equal to 0 were excluded; * regions in bold present the π value for regions with more than one sequence in a raw presenting high π values.

Supplementary Tables

Table S1. Codon usage for *Byrsonima coccolobifolia* and *B. crassifolia*

Codon	Amino acid	Number of codons		Codon	Amino acid	Number of codons	
		B. coccolobifolia	*B. crassifolia*			*B. coccolobifolia*	*B. crassifolia*
GCA	A	413	414	CCA	P	310	312
GCC	A	241	239	CCC	P	217	219
GCG	A	153	155	CCG	P	157	156
GCT	A	667	667	CCT	P	450	450
GC	C	106	106	CAA	Q	775	772
TGT	C	236	237	CAG	Q	230	233
GAC	D	245	246	AGA	R	531	531
GAT	D	904	905	AGG	R	192	193
GAA	E	1126	1121	CGA	R	378	379
GAG	E	387	386	CGC	R	114	114
TTC	F	583	579	CGG	R	122	123
TTT	F	1127	1126	CGT	R	351	350
GGA	G	772	771	AGC	S	156	155
GGC	G	194	193	AGT	S	437	436
GGG	G	332	334	TCA	S	444	445
GGT	G	630	628	TCC	S	381	383
CAC	H	170	169	TCG	S	203	204
CAT	H	556	555	TCT	S	641	638
ATA	I	785	784	ACA	T	438	441
ATC	I	468	466	ACC	T	279	277
ATT	I	1210	1213	ACG	T	157	158
AAA	K	1145	1145	ACT	T	566	566
AAG	K	385	385	GTA	V	557	559
CTA	L	432	432	GTC	V	189	191
CTC	L	210	213	GTG	V	231	225
CTG	L	199	200	GTT	V	534	535
CTT	L	641	639	TGG	W	538	537
TTA	L	949	946	TAC	Y	194	193
TTG	L	595	596	TAT	Y	854	855
ATG	M	654	654	TAA	*	64	63
AAC	N	329	327	TAG	*	37	37
AAT	N	1102	1105	TGA	*	29	29

Table S2. Evolutionary rates of eighty six gene sequences between the chloroplast genomes of nine species of the order Malpighiales (for species abbreviation see Supplementary Table S1).

Genes	Species	Sequence lenght	Ka	Ks	Ka/Ks
	Bc	1059	0.0067	0.2234	0.0302
	Br	1059	0.0067	0.2329	0.0289
	Ci	1059	0.0061	0.2542	0.0239
	Hr	1059	0.0062	0.2483	0.0251
**psbA**	Vs	1059	0.0070	0.2568	0.0273
	Pa	1059	0.0039	0.2652	0.0147
	Sp	1059	0.0064	0.2544	0.0251
	Me	1059	0.0020	0.1724	0.0117
	Rc	1059	0.0022	0.2040	0.0107
	Bc	1509	0.1610	0.3463	0.4649
	Br	1503	0.1564	0.3363	0.4649
	Ci	1191	0.1698	0.4740	0.3583
	Hr	729	0.1516	0.4564	0.3322
**matK**	Vs	1356	0.1839	0.4611	0.3988
	Pa	1506	0.1554	0.3339	0.4654
	Sp	1500	0.1469	0.3542	0.4148
	Me	1386	0.1732	0.1103	1.5700
	Rc	1509	0.1431	0.3398	0.4213
	Bc	213	0.0736	0.3719	0.1979
	Br	216	0.0972	0.3560	0.2731
	Ci	204	0.1921	0.3620	0.5306
	Hr	204	0.1700	0.3715	0.4574
**rps16**	Vs	-	-	-	-
	Pa	-	-	-	-
	Sp	-	-	-	-
	Me	216	0.0607	0.2167	0.2802

	Rc	216	0.0657	0.2748	0.2390
psbK	Bc	183	0.0301	0.4229	0.0713
	Br	183	0.0302	0.4229	0.0713
	Ci	183	0.0475	0.4188	0.1134
	Hr	183	0.0446	0.4150	0.1076
	Vs	171	0.0301	0	0.0629
	Pa	183	0.0314	0.4361	0.0721
	Sp	183	0.0273	0.4301	0.0636
	Me	183	0.0480	0.6296	0.0763
	Rc	183	0.0333	0.8613	0.0387
psbI	Bc	108	0.0479	0.6417	0.0746
	Br	108	0.0479	0.6417	0.0746
	Ci	123	0.0092	0.3329	0.0276
	Hr	120	0.0003	0.3629	0.0009
	Vs	129	0.0257	0.3667	0.0700
	Pa	108	0.0005	0.4988	0.0010
	Sp	135	0.0233	0.3914	0.0595
	Me	153	0.0854	0.1680	0.5086
	Rc	132	0.0312	0.2484	0.1258
atpA	Bc	1515	0.0368	0.3044	0.1211
	Br	1515	0.0370	0.3026	0.1222
	Ci	1518	0.0457	0.3450	0.1324
	Hr	1518	0.0483	0.3498	0.1381
	Vs	1515	0.0582	0.4227	0.1377
	Pa	1518	0.0567	0.3217	0.1764
	Sp	1518	0.0672	0.3397	0.1979
	Me	1518	0.0378	0.2862	0.1322
	Rc	1518	0.0349	0.3109	0.1123
atpF	Bc	552	0.0842	0.5309	0.1586

	Br	552	0.0842	0.5309	0.1586
	Ci	549	0.1069	0.6690	0.1598
	Hr	564	0.0938	0.6617	0.1418
	Vs	564	0.0784	0.5975	0.1312
	Pa	627	0.0513	0.4640	0.1106
	Sp	561	0.0487	0.5145	0.0947
	Me	552	0.0827	0.4819	0.1715
	Rc	612	0.0818	0.4845	0.1689
**atpH**	Bc	243	0.0001	0.1645	0.0010
	Br	243	0.0002	0.1646	0.0010
	Ci	243	0.0003	0.2306	0.0014
	Hr	243	0.0003	0.2306	0.0014
	Vs	243	0.0035	0.1862	0.0188
	Pa	243	0.0002	0.1254	0.0016
	Sp	243	0.0002	0.1412	0.0014
	Me	243	0.0003	0.2051	0.0013
	Rc	243	0.0002	0.1499	0.0013
**atpI**	Bc	741	0.0206	0.2457	0.0840
	Br	741	0.0206	0.2457	0.0840
	Ci	741	0.0203	0.6437	0.0315
	Hr	741	0.0187	0.6359	0.0294
	Vs	741	0.0251	0.5657	0.0444
	Pa	741	0.0268	0.4266	0.0629
	Sp	741	0.0258	0.4142	0.0624
	Me	741	0.0173	0.4664	0.0371
	Rc	741	0.0172	0.4806	0.0358
**rps2**	Bc	705	0.0472	0.2965	0.1591
	Br	705	0.0472	0.2965	0.1591
	Ci	708	0.0528	0.3456	0.1529

	Hr	708	0.0502	0.3542	0.1418
	Vs	705	0.0687	0.3736	0.1839
	Pa	708	0.0642	0.2718	0.2363
	Sp	708	0.0615	0.2707	0.2272
	Me	708	0.0458	0.2534	0.1806
	Rc	708	0.0392	0.2810	0.1395
**rpoC2**	Bc	4143	0.0919	0.2828	0.3251
	Br	4143	0.0917	0.2806	0.3269
	Ci	4152	0.0978	0.2857	0.3425
	Hr	4140	0.0724	0.2801	0.2584
	Vs	3759	0.0936	0.3434	0.2725
	Pa	4137	0.0868	0.2836	0.3061
	Sp	4104	0.0870	0.3058	0.2846
	Me	4089	0.0724	0.2801	0.2584
	Rc	4143	0.0799	0.2705	0.2955
**rpoC1**	Bc	2052	0.0444	0.2811	0.1582
	Br	2037	0.0457	0.2751	0.1664
	Ci	2049	0.0380	0.2561	0.1483
	Hr	2049	0.0383	0.2618	0.1463
	Vs	2046	0.0491	0.2815	0.1743
	Pa	2052	0.0407	0.2551	0.1597
	Sp	2061	0.0406	0.2666	0.1524
	Me	2052	0.0319	0.2354	0.1353
	Rc	2052	0.0342	0.2390	0.1430
**rpoB**	Bc	3210	0.0378	0.2757	0.1370
	Br	3210	0.0377	0.2737	0.1378
	Ci	3210	0.0448	0.2924	0.1532
	Hr	3210	0.0440	0.3062	0.1437
	Vs	3207	0.0483	0.3056	0.1582

	Pa	3207	0.0426	0.2595	0.1643
	Sp	3207	0.0434	0.2623	0.1656
	Me	3210	0.0352	0.2380	0.1480
	Rc	3210	0.0341	0.2501	0.1363
**petN**	Bc	93	0.0307	0.0905	0.3397
	Br	93	0.0307	0.0905	0.3397
	Ci	93	0.0006	0.1391	0.0042
	Hr	93	0.0006	0.1391	0.0042
	Vs	93	0.0004	0.0917	0.0043
	Pa	93	0.0006	0.1123	0.0052
	Sp	93	0.0006	0.1123	0.0052
	Me	93	0.0004	0.0798	0.0049
	Rc	93	0.0008	0.1746	0.0045
**psbM**	Bc	102	0.0114	0.3785	0.0302
	Br	102	0.0114	0.3785	0.0302
	Ci	102	0.0486	0.3034	0.1603
	Hr	102	0.0095	0.3458	0.0274
	Vs	102	0.0319	0.2392	0.1334
	Pa	102	0.0086	0.5573	0.0155
	Sp	102	0.0086	0.5573	0.0155
	Me	102	0.0193	0.2635	0.0730
	Rc	102	0.0109	0.3558	0.0306
**psbD**	Bc	1059	0.0025	0.2513	0.0102
	Br	1059	0.0038	0.2532	0.0152
	Ci	1059	0.0114	0.3742	0.0304
	Hr	1059	0.0114	0.3597	0.0317
	Vs	1059	0.0070	0.3814	0.0184
	Pa	1059	0.0076	0.3028	0.0251
	Sp	1059	0.0038	0.3284	0.0116

	Me	1059	0.0006	0.2310	0.0025
	Rc	1059	0.0006	0.2255	0.0026
**psbC**	Bc	1458	0.0068	0.2040	0.0333
	Br	1458	0.0068	0.2040	0.0333
	Ci	1458	0.0091	0.2438	0.0372
	Hr	1458	0.0091	0.2360	0.0384
	Vs	1458	0.0094	0.2611	0.0362
	Pa	1458	0.0080	0.1997	0.0400
	Sp	1458	0.0091	0.2149	0.0422
	Me	1458	0.0058	0.1901	0.0306
	Rc	1458	0.0057	0.2026	0.0281
**psbZ**	Bc	186	0.0299	0.7953	0.0376
	Br	186	0.0299	0.7953	0.0376
	Ci	186	0.0392	0.5982	0.0656
	Hr	186	0.0307	0.6084	0.0505
	Vs	186	0.0222	0.9505	0.0234
	Pa	186	0.0229	0.7278	0.0315
	Sp	186	0.0233	0.9463	0.0246
	Me	186	0.0226	0.5465	0.0413
	Rc	186	0.0310	0.6639	0.0466
**rps14**	Bc	300	0.0713	0.1124	0.6343
	Br	300	0.0713	0.1124	0.6343
	Ci	300	0.0573	0.1103	0.5196
	Hr	300	0.0580	0.1194	0.4855
	Vs	300	0.0646	0.2125	0.3037
	Pa	300	0.0336	0.1459	0.2305
	Sp	300	0.0330	0.1826	0.1804
	Me	300	0.0336	0.1270	0.2648
	Rc	300	0.0264	0.1429	0.1845

**psaB**	Bc	2202	0.0134	0.3832	0.0350
	Br	2202	0.0134	0.3832	0.0350
	Ci	2202	0.0111	0.3344	0.0332
	Hr	2202	0.0123	0.3260	0.0378
	Vs	2202	0.0138	0.3803	0.0362
	Pa	2202	0.0112	0.2818	0.0398
	Sp	2202	0.0111	0.3265	0.0340
	Me	2202	0.0122	0.3186	0.0383
	Rc	2202	0.0120	0.3243	0.0370
**psaA**	Bc	2250	0.0085	0.3157	0.0269
	Br	2250	0.0085	0.3157	0.0269
	Ci	2250	0.0077	0.3042	0.0254
	Hr	2250	0.0086	0.3021	0.0285
	Vs	2250	0.0115	0.3075	0.0375
	Pa	2250	0.0101	0.2759	0.0365
	Sp	2250	0.0102	0.2738	0.0373
	Me	2250	0.0060	0.2545	0.0235
	Rc	2250	0.0096	0.2818	0.0342
**ycf3**	Bc	501	0.0180	0.2372	0.0761
	Br	504	0.0179	0.2366	0.0758
	Ci	504	0.0147	0.3406	0.0433
	Hr	504	0.0178	0.3290	0.0542
	Vs	375	0.0081	0.4931	0.0165
	Pa	504	0.0144	0.2771	0.0520
	Sp	504	0.0200	0.2665	0.0751
	Me	504	0.0151	0.3155	0.0478
	Rc	504	0.0136	0.2583	0.0528
**rps4**	Bc	603	0.0634	0.2262	0.2803
	Br	603	0.0605	0.2290	0.2641

	Ci	603	0.0805	0.1999	0.4024
	Hr	603	0.0794	0.1943	0.4083
	Vs	603	0.0729	0.1925	0.3785
	Pa	603	0.0545	0.1690	0.3226
	Sp	603	0.0577	0.1765	0.3269
	Me	603	0.0484	0.1888	0.2565
	Rc	603	0.0542	0.1856	0.2922
**ndhJ**	Bc	474	0.0279	0.1720	0.1621
	Br	474	0.0286	0.1817	0.1571
	Ci	474	0.0216	0.1853	0.1166
	Hr	474	0.0198	0.1743	0.1138
	Vs	474	0.0209	0.2197	0.0953
	Pa	471	0.0207	0.1837	0.1128
	Sp	471	0.0197	0.2110	0.0933
	Me	474	0.0196	0.1817	0.1080
	Rc	474	0.0249	0.1996	0.1250
**ndhK**	Bc	669	0.0409	0.4929	0.0830
	Br	816	0.0420	0.4638	0.0905
	Ci	843	0.0481	0.3855	0.1248
	Hr	846	0.0466	0.3794	0.1228
	Vs	843	0.0559	0.3976	0.1405
	Pa	852	0.0451	0.2980	0.1513
	Sp	852	0.0450	0.3274	0.1375
	Me	819	0.0456	0.3150	0.1449
	Rc	846	0.0506	0.2465	0.2053
**ndhC**	Bc	357	0.0484	0.3930	0.1233
	Br	357	0.0484	0.3930	0.1233
	Ci	360	0.0760	0.6803	0.1117
	Hr	360	0.0650	0.4356	0.1493

	Vs	360	0.0739	0.5236	0.1411
	Pa	360	0.0551	0.8256	0.0667
	Sp	360	0.0509	0.8257	0.0616
	Me	360	0.0731	0.4034	0.1812
	Rc	360	0.0660	0.4913	0.1344
**atpE**	Bc	396	0.3333	0.2154	1.5471
	Br	396	0.3340	0.2053	1.6269
	Ci	396	0.0386	0.1913	0.2018
	Hr	396	0.0386	0.1913	0.2018
	Vs	399	0.0409	0.1911	0.2141
	Pa	396	0.0341	0.2118	0.1609
	Sp	396	0.0368	0.2110	0.1744
	Me	399	0.0280	0.1967	0.1422
	Rc	396	0.0383	0.1872	0.2043
**atpB**	Bc	1494	0.0301	0.3375	0.0893
	Br	1494	0.0301	0.3425	0.0878
	Ci	1494	0.0289	0.4139	0.0699
	Hr	1494	0.0280	0.4166	0.0671
	Vs	1494	0.0351	0.4505	0.0779
	Pa	1494	0.0284	0.3217	0.0884
	Sp	1494	0.0304	0.3329	0.0912
	Me	1491	0.0255	0.2825	0.0904
	Rc	1494	0.0248	0.3483	0.0712
**rbcL**	Bc	1425	0.0192	0.2792	0.0689
	Br	1425	0.0165	0.2773	0.0598
	Ci	1425	0.0144	0.3625	0.0397
	Hr	1425	0.0150	0.3854	0.0390
	Vs	1425	0.0171	0.4335	0.0395
	Pa	1425	0.0154	0.2843	0.0541

	Sp	1431	0.0155	0.3118	0.0497
	Me	1431	0.0161	0.2815	0.0573
	Rc	1425	0.0150	0.2983	0.0504
**accD**	Bc	1470	0.1552	0.4397	0.3530
	Br	924	0.0807	0.3549	0.2274
	Ci	1458	0.1685	0.4095	0.4115
	Hr	1443	0.1727	0.3950	0.4373
	Vs	1245	0.1131	0.5223	0.2165
	Pa	1467	0.1620	0.3450	0.4696
	Sp	1458	0.1645	0.3702	0.4445
	Me	1233	0.1117	0.3542	0.3153
	Rc	1500	0.1557	0.2964	0.5252
**psaI**	Bc	105	0.0560	0.0703	0.7975
	Br	105	0.0560	0.0703	0.7975
	Ci	108	0.0752	0.1414	0.5320
	Hr	108	0.0752	0.1414	0.5320
	Vs	108	0.0565	0.5684	0.0994
	Pa	105	0.0549	0.5502	0.0998
	Sp	105	0.0549	0.5502	0.0998
	Me	105	0.0549	0.6828	0.0804
	Rc	105	0.0599	0.1786	0.3355
**ycf4**	Bc	552	0.0314	0.1896	0.1659
	Br	552	0.0314	0.1896	0.1659
	Ci	552	0.0562	0.2357	0.2385
	Hr	552	0.0563	0.2266	0.2484
	Vs	549	0.0462	0.2505	0.1844
	Pa	552	0.0377	0.1690	0.2230
	Sp	552	0.0412	0.1768	0.2328
	Me	552	0.0415	0.2034	0.2040

	Rc	552	0.0362	0.2075	0.1744
**cemA**	Bc	684	0.1297	0.1936	0.6700
	Br	684	0.1247	0.2025	0.6159
	Ci	687	0.1415	0.2192	0.6456
	Hr	681	0.1376	0.2520	0.5462
	Vs	684	0.1244	0.2827	0.4400
	Pa	681	0.1250	0.2342	0.5336
	Sp	681	0.1347	0.2159	0.6238
	Me	678	0.1229	0.2795	0.4396
	Rc	681	0.1255	0.2780	0.4515
**petA**	Bc	960	0.0424	0.3453	0.1230
	Br	960	0.0442	0.3505	0.1263
	Ci	960	0.0385	0.3149	0.1224
	Hr	960	0.0368	0.3298	0.1117
	Vs	960	0.0463	0.3899	0.1187
	Pa	960	0.0402	0.2581	0.1558
	Sp	960	0.0386	0.3015	0.1282
	Me	960	0.0315	0.2446	0.1287
	Rc	960	0.0348	0.2614	0.1332
**psbJ**	Bc	120	0.0485	0.2212	0.2196
	Br	120	0.0485	0.2212	0.2196
	Ci	120	0.0153	0.7048	0.0218
	Hr	120	0.0165	0.6239	0.0264
	Vs	120	0.0308	0.2990	0.1031
	Pa	120	0.0290	0.4687	0.0618
	Sp	120	0.0294	0.3595	0.0818
	Me	120	0.0271	0.4358	0.0622
	Rc	120	0.0142	0.4540	0.0313
**psbL**	Bc	114	0.0001	0.1697	0.0010

	Br	114	0.0001	0.1697	0.0010
	Ci	114	0.0006	0.7364	0.0009
	Hr	114	0.0006	0.7364	0.0009
	Vs	114	0.0010	0.3733	0.0027
	Pa	114	0.0011	0.8262	0.0013
	Sp	114	0.0011	0.8249	0.0014
	Me	114	0.0010	0.3707	0.0028
	Rc	114	0.0009	0.1694	0.0056
**psbF**	Bc	117	0.0120	0.2301	0.0522
	Br	117	0.0120	0.2301	0.0522
	Ci	117	0.0001	0.1834	0.0008
	Hr	117	0.0001	0.1834	0.0008
	Vs	117	0.0061	0.1828	0.0333
	Pa	117	0.0056	0.1709	0.0329
	Sp	117	0.0056	0.1709	0.0329
	Me	117	0.0057	0.1039	0.0550
	Rc	117	0.0057	0.1043	0.0543
**psbE**	Bc	249	0.0103	0.2955	0.0351
	Br	249	0.0103	0.2955	0.0351
	Ci	249	0.0112	0.3986	0.0282
	Hr	249	0.0142	0.3757	0.0378
	Vs	249	0.0121	0.4978	0.0243
	Pa	249	0.0005	0.3350	0.0016
	Sp	249	0.0116	0.3251	0.0355
	Me	249	0.0060	0.5026	0.0120
	Rc	249	0.0055	0.3970	0.0139
**petL**	Bc	93	0.0775	0.1091	0.7106
	Br	93	0.0775	0.1091	0.7106
	Ci	93	0.0878	0.5650	0.1554

	Hr	93	0.0878	0.5650	0.1554
	Vs	93	0.1170	0.8299	0.1410
	Pa	93	0.1620	0.7745	0.2092
	Sp	93	0.2237	0.6629	0.3375
	Me	93	0.0505	0.1082	0.4665
	Rc	93	0.0234	0.1046	0.2239
**petG**	Bc	111	0.0134	0.1601	0.0836
	Br	111	0.0134	0.1601	0.0836
	Ci	111	0.0134	0.1689	0.0794
	Hr	111	0.0134	0.2135	0.0628
	Vs	111	0.0335	0.5444	0.0615
	Pa	111	0.0134	0.2616	0.0512
	Sp	111	0.0201	0.3325	0.0604
	Me	111	0.0134	0.1646	0.0814
	Rc	111	0.0122	0.1284	0.0950
**psaJ**	Bc	123	0.0816	0.5359	0.1523
	Br	123	0.0816	0.5359	0.1523
	Ci	132	0.0254	0.8005	0.0317
	Hr	132	0.0254	0.8005	0.0317
	Vs	132	0.0529	0.2687	0.1967
	Pa	132	0.0582	0.4303	0.1353
	Sp	132	0.0499	0.3411	0.1464
	Me	132	0.0507	0.3314	0.1531
	Rc	132	0.0809	0.2266	0.3570
**rpl33**	Bc	198	0.0639	0.4228	0.1513
	Br	198	0.0639	0.4228	0.1513
	Ci	198	0.0816	0.3656	0.2232
	Hr	198	0.0816	0.3656	0.2232
	Vs	198	0.0797	0.3284	0.2426

	Pa	198	0.0765	0.6341	0.1207
	Sp	198	0.0789	0.5649	0.1397
	Me	198	0.0962	0.5047	0.1907
	Rc	198	0.0591	0.5266	0.1122
**rps18**	Bc	300	0.1143	0.3007	0.3800
	Br	300	0.1143	0.3007	0.3800
	Ci	258	0.0906	0.3185	0.2844
	Hr	258	0.0906	0.3185	0.2844
	Vs	258	0.0897	0.2339	0.3837
	Pa	300	0.1226	0.1815	0.6756
	Sp	300	0.1219	0.1515	0.8047
	Me	258	0.0497	0.1735	0.2865
	Rc	258	0.0409	0.1950	0.2096
**rpl20**	Bc	366	0.1498	0.3277	0.4571
	Br	366	0.1498	0.3277	0.4571
	Ci	366	0.1234	0.3745	0.3295
	Hr	366	0.1337	0.3271	0.4089
	Vs	348	0.1478	0.4359	0.3390
	Pa	369	0.1377	0.3233	0.4258
	Sp	369	0.1492	0.3488	0.4278
	Me	351	0.0743	0.3775	0.1969
	Rc	375	0.1035	0.3802	0.2723
**rps12**	Bc	114	0.0932	0.1870	0.4987
	Br	114	0.0932	0.1870	0.4987
	Ci	114	0.0903	0.3023	0.2986
	Hr	114	0.0903	0.3023	0.2986
	Vs	114	0.0925	0.3112	0.2971
	Pa	114	0.0939	0.3119	0.3011
	Sp	114	0.0939	0.3119	0.3011

	Me	114	0.0932	0.1863	0.5003
	Rc	114	0.1805	0.2030	0.8895
**clpP**	Bc	582	0.0660	0.2564	0.2574
	Br	579	0.0636	0.2578	0.2467
	Ci	579	0.0642	0.2534	0.2536
	Hr	579	0.0639	0.2629	0.2431
	Vs	1680	0.2536	0.1373	1.8474
	Pa	579	0.0820	0.2030	0.4038
	Sp	522	0.0815	0.2350	0.3469
	Me	579	0.0714	0.2528	0.2824
	Rc	579	0.0732	0.2451	0.2985
**psbB**	Bc	1518	0.0101	0.3807	0.0266
	Br	1518	0.0101	0.3807	0.0266
	Ci	1518	0.0051	0.4283	0.0119
	Hr	1518	0.0062	0.4215	0.0147
	Vs	1518	0.0073	0.5720	0.0128
	Pa	1518	0.0085	0.3703	0.0229
	Sp	1518	0.0090	0.3710	0.0244
	Me	1518	0.0041	0.3443	0.0118
	Rc	1518	0.0062	0.4323	0.0144
**psbT**	Bc	114	0.0135	0.1883	0.0717
	Br	108	0.0001	0.1548	0.0010
	Ci	108	0.0122	0.5445	0.0224
	Hr	108	0.0002	0.3275	0.0006
	Vs	108	0.0001	0.2516	0.0004
	Pa	108	0.0002	0.3374	0.0006
	Sp	108	0.0003	0.5618	0.0005
	Me	108	0.0123	0.6944	0.0177
	Rc	108	0.0130	0.7906	0.0164

	Bc	129	0.0001	0.1192	0.0010
	Br	129	0.0001	0.1192	0.0010
	Ci	129	0.0001	0.2027	0.0005
	Hr	129	0.0001	0.2027	0.0005
**psbN**	Vs	129	0.0001	0.0960	0.0011
	Pa	129	0.0001	0.0954	0.0011
	Sp	129	0.0001	0.1244	0.0008
	Me	129	0.0001	0.0954	0.0011
	Rc	129	0.0000	0.0979	0.0002
	Bc	219	0.0250	0.2963	0.0845
	Br	219	0.0250	0.2963	0.0845
	Ci	219	0.0442	0.2705	0.1635
	Hr	219	0.0442	0.2705	0.1635
**psbH**	Vs	219	0.0482	0.2026	0.2377
	Pa	219	0.0210	0.1826	0.1150
	Sp	219	0.0216	0.2234	0.0965
	Me	219	0.0258	0.2576	0.1003
	Rc	219	0.0165	0.2516	0.0654
	Bc	645	0.0083	0.3268	0.0255
	Br	645	0.0083	0.3268	0.0255
	Ci	645	0.0073	0.5609	0.0131
	Hr	645	0.0073	0.5835	0.0126
**petB**	Vs	345	0.0075	0.5539	0.0135
	Pa	645	0.0101	0.3909	0.0258
	Sp	645	0.0048	0.4235	0.0114
	Me	645	0.0052	0.3576	0.0147
	Rc	645	0.0055	0.3193	0.0172
**petD**	Bc	522	0.0097	0.2354	0.0411
	Br	522	0.0072	0.2404	0.0301

	Ci	522	0.0115	0.3139	0.0365
	Hr	522	0.0118	0.2748	0.0428
	Vs	522	0.0095	0.2358	0.0403
	Pa	522	0.0095	0.1990	0.0478
	Sp	522	0.0096	0.2175	0.0442
	Me	522	0.0053	0.2626	0.0201
	Rc	522	0.0077	0.2345	0.0330
**rpoA**	Bc	999	0.0689	0.4018	0.1714
	Br	987	0.0719	0.3832	0.1877
	Ci	1005	0.0655	0.3661	0.1788
	Hr	1005	0.0651	0.4037	0.1612
	Vs	972	0.0632	0.4257	0.1485
	Pa	1008	0.0613	0.2640	0.2324
	Sp	1008	0.0574	0.2728	0.2105
	Me	1002	0.0534	0.3099	0.1723
	Rc	975	0.0570	0.2733	0.2085
**rps11**	Bc	414	0.1138	0.3385	0.3361
	Br	405	0.1291	0.4439	0.2909
	Ci	414	0.1315	0.3805	0.3456
	Hr	414	0.1315	0.3638	0.3615
	Vs	414	0.1153	0.3484	0.3309
	Pa	414	0.1289	0.4892	0.2635
	Sp	414	0.1286	0.4716	0.2727
	Me	414	0.0931	0.3415	0.2725
	Rc	414	0.1000	0.3390	0.2950
**rpl36**	Bc	111	0.0915	0.9150	0.1000
	Br	111	0.0915	0.9150	0.1000
	Ci	111	0.1300	0.7194	0.1807
	Hr	111	0.1897	0.7274	0.2607

	Vs	111	0.0023	0.5700	0.0041
	Pa	111	0.1752	0.4264	0.4110
	Sp	111	0.0555	0.5365	0.1034
	Me	111	0.0601	0.6598	0.0911
	Rc	111	0.0662	1.1525	0.0574
	Bc	-	-	-	-
	Br	-	-	-	-
	Ci	81	0.2043	0.7765	0.2631
	Hr	102	0.2192	0.6432	0.3408
**infA**	Vs	-	-	-	-
	Pa	156	0.3504	0.2735	1.2809
	Sp	159	0.3497	0.3877	0.9019
	Me	-	-	-	-
	Rc	54	0.3865	0.7774	0.4972
	Bc	402	0.1035	0.4594	0.2253
	Br	402	0.1035	0.4594	0.2253
	Ci	402	0.1283	0.6471	0.1982
	Hr	402	0.1230	0.5648	0.2177
**rps8**	Vs	402	0.1161	0.4936	0.2352
	Pa	402	0.1048	0.4006	0.2615
	Sp	402	0.1025	0.4234	0.2420
	Me	402	0.0942	0.6259	0.1506
	Rc	402	0.0936	0.5946	0.1573
	Bc	366	0.0989	0.3345	0.2957
	Br	366	0.0989	0.3345	0.2957
**rpl14**	Ci	366	0.1854	0.3859	0.4803
	Hr	366	0.1861	0.3880	0.4797
	Vs	366	0.1749	0.3908	0.4476
	Pa	366	0.1175	0.2951	0.3981

	Sp	366	0.1216	0.3497	0.3477
	Me	366	0.1044	0.2228	0.4688
	Rc	366	0.0936	0.2506	0.3737
**rpl16**	Bc	396	0.0423	0.2850	0.1486
	Br	396	0.0423	0.2850	0.1486
	Ci	396	0.0385	0.2742	0.1404
	Hr	396	0.0382	0.2971	0.1285
	Vs	396	0.0501	0.3047	0.1643
	Pa	393	0.0459	0.2143	0.2140
	Sp	393	0.0497	0.2170	0.2291
	Me	396	0.0330	0.2701	0.1222
	Rc	396	0.0299	0.2304	0.1299
**rps3**	Bc	654	0.1132	0.4355	0.2600
	Br	654	0.1177	0.4364	0.2697
	Ci	654	0.1855	0.5303	0.3498
	Hr	654	0.1873	0.5064	0.3698
	Vs	651	0.1381	0.5707	0.2420
	Pa	654	0.1711	0.5521	0.3098
	Sp	654	0.1771	0.5929	0.2987
	Me	654	0.1161	0.5340	0.2174
	Rc	654	0.1152	0.5967	0.1930
**rpl22**	Bc	387	0.1553	0.5810	0.2674
	Br	378	0.1218	0.4816	0.2528
	Ci	402	0.1789	0.6641	0.2693
	Hr	381	0.1157	0.6330	0.1828
	Vs	435	0.1425	0.6519	0.2185
	Pa	363	0.1364	0.4166	0.3274
	Sp	417	0.1801	0.3607	0.4992
	Me	378	0.1093	0.5044	0.2168

	Rc	276	0.1801	0.3607	0.4992
**rps19**	Bc	276	0.0319	0.5732	0.0557
	Br	273	0.0322	0.5658	0.0570
	Ci	276	0.0446	0.5681	0.0784
	Hr	276	0.0446	0.5681	0.0784
	Vs	276	0.0530	0.8429	0.0629
	Pa	276	0.0474	0.7993	0.0593
	Sp	276	0.0476	0.8433	0.0565
	Me	276	0.0249	0.5242	0.0474
	Rc	276	0.0199	0.4006	0.0497
**rpl2**	Bc	819	0.0173	0.0691	0.2504
	Br	822	0.0153	0.0705	0.2177
	Ci	663	0.0240	0.0867	0.2769
	Hr	666	0.0239	0.0869	0.2746
	Vs	645	0.0253	0.1081	0.2339
	Pa	666	0.0267	0.0699	0.3821
	Sp	663	0.0212	0.0696	0.3042
	Me	666	0.0246	0.0602	0.4078
	Rc	666	0.0246	0.0592	0.4152
**rpl23**	Bc	270	0.0461	0.0528	0.8734
	Br	270	0.0461	0.0528	0.8734
	Ci	279	0.0349	0.0807	0.4328
	Hr	279	0.0274	0.0800	0.3419
	Vs	279	0.0266	0.0525	0.5076
	Pa	279	0.0410	0.0640	0.6417
	Sp	279	0.0497	0.0618	0.8035
	Me	279	0.0270	0.0518	0.5210
	Rc	279	0.0265	0.0868	0.3050
**ycf2**	Bc	6801	0.0437	0.0642	0.6805

	Br	6804	0.0437	0.0633	0.6902
	Ci	6801	0.0435	0.0572	0.7603
	Hr	6819	0.0452	0.0580	0.7800
	Vs	6753	0.0496	0.0615	0.8071
	Pa	6789	0.0399	0.0576	0.6932
	Sp	6783	0.0408	0.0633	0.6450
	Me	6726	0.0422	0.0500	0.8441
	Rc	6810	0.0415	0.0549	0.7554
**ycf15**	Bc	150	0.8765	0.8014	1.0938
	Br	144	0.8257	0.7360	1.1218
	Ci	198	0.4305	0.5894	0.7305
	Hr	162	0.2320	0.4257	0.5450
	Vs	165	0.3355	0.1956	1.7155
	Pa	198	0.3078	0.2066	1.4901
	Sp	180	0.3150	0.2120	1.4864
	Me	177	0.3683	0.2847	1.2937
	Rc	198	0.3375	0.6116	0.5518
**ndhB**	Bc	1530	0.0132	0.0286	0.4642
	Br	1530	0.0132	0.0286	0.4642
	Ci	1530	0.0139	0.0350	0.3971
	Hr	1530	0.0139	0.0331	0.4201
	Vs	1530	0.0144	0.0279	0.5149
	Pa	1530	0.0129	0.0300	0.4299
	Sp	1530	0.0128	0.0316	0.4045
	Me	465	0.0100	0.0280	0.3578
	Rc	1530	0.0085	0.0270	0.3152
**rps7**	Bc	465	0.0095	0.0555	0.1709
	Br	465	0.0109	0.0554	0.1982
	Ci	465	0.0041	0.0456	0.0904

	Hr	465	0.0041	0.0456	0.0904
	Vs	465	0.0096	0.0299	0.3196
	Pa	465	0.0094	0.0386	0.2439
	Sp	306	0.0162	0.0540	0.2997
	Me	465	0.0054	0.0303	0.1790
	Rc	465	0.0082	0.0297	0.2765
**rps12_3end**	Bc	249	0.0001	0.0175	0.0010
	Br	249	0.0001	0.0175	0.0010
	Ci	249	0.0001	0.0275	0.0038
	Hr	249	0.0001	0.0275	0.0038
	Vs	249	0.0001	0.0287	0.0043
	Pa	249	0.0001	0.0274	0.0038
	Sp	249	0.0001	0.0078	0.0169
	Me	249	0.0002	0.0185	0.0108
	Rc	249	0.0001	0.0293	0.0038
**rps12**	Bc	240	0.0001	0.0181	0.0010
	Br	240	0.0001	0.0181	0.0010
	Ci	240	0.0001	0.0281	0.0033
	Hr	240	0.0001	0.0281	0.0033
	Vs	240	0.0001	0.0296	0.0036
	Pa	240	0.0001	0.0281	0.0033
	Sp	240	0.0001	0.0081	0.0143
	Me	240	0.0001	0.0191	0.0052
	Rc	240	0.0001	0.0301	0.0032
**ycf68**	Bc	159	0.0543	0.0545	0.9980
	Br	159	0.0406	0.0446	0.9093
	Ci	165	0.0680	0.0197	3.4490
	Hr	165	0.0584	0.0573	1.0188
	Vs	162	0.0098	0.0219	0.4476

	Pa	57	0.1918	0.8331	0.2302
	Sp	165	0.0274	0.0451	0.6075
	Me	168	0.0201	0.0364	0.5536
	Rc	57	0.0203	0.7823	0.0259
**orf42**	Bc	120	0.0459	0.0872	0.5264
	Br	120	0.0459	0.0872	0.5264
	Ci	120	0.1457	0.0727	2.0044
	Hr	120	0.1457	0.0727	2.0044
	Vs	120	0.1343	0.0902	1.4884
	Pa	120	0.0973	0.0742	1.3102
	Sp	120	0.1461	0.0718	2.0354
	Me	120	0.0510	0.1626	0.3137
	Rc	120	0.0510	0.1626	0.3137
**orf56**	Bc	60	0.0942	0.1156	0.8149
	Br	54	0.0643	0.1647	0.3906
	Ci	72	0.0456	0.1170	0.3898
	Hr	72	0.0456	0.1170	0.3898
	Vs	72	0.0219	0.1171	0.1870
	Pa	72	0.0219	0.1171	0.1870
	Sp	72	0.0456	0.1173	0.3888
	Me	69	0.0222	0.1286	0.1728
	Rc	66	0.0218	0.0660	0.3297
**ycf1**	Bc	984	0.0801	0.0722	1.1094
	Br	984	0.0787	0.0716	1.0992
	Ci	984	0.0839	0.1047	0.8019
	Hr	978	0.0809	0.1053	0.7678
	Vs	5151	0.2827	0.4851	0.5828
	Pa	975	0.0613	0.0596	1.0284
	Sp	969	0.0561	0.0705	0.7961

	Me	966	0.0616	0.0658	0.9363
	Rc	969	0.0728	0.0734	0.9928
**ndhF**	Bc	2208	0.1208	0.8423	0.1434
	Br	2136	0.1474	0.4361	0.3380
	Ci	2091	0.1498	0.6895	0.2173
	Hr	2073	0.1339	0.7426	0.1803
	Vs	2181	0.1372	0.6870	0.1997
	Pa	2133	0.1170	0.6696	0.1748
	Sp	2205	0.1288	0.7228	0.1782
	Me	2133	0.1136	0.6134	0.1852
	Rc	2196	0.1251	0.6120	0.2044
**rpl32**	Bc	129	0.1032	0.4613	0.2237
	Br	149	0.1208	0.5008	0.2412
	Ci	159	0.0608	0.6309	0.0964
	Hr	159	0.0976	0.5969	0.1635
	Vs	-	-	-	-
	Pa	-	-	-	-
	Sp	-	-	-	-
	Me	162	0.0829	0.5463	0.1518
	Rc	165	0.1034	0.5701	0.1814
**ccsA**	Bc	927	0.1356	0.4433	0.3060
	Br	930	0.1346	0.4582	0.2938
	Ci	915	0.1587	0.4180	0.3797
	Hr	915	0.1635	0.4289	0.3811
	Vs	933	0.1559	0.4829	0.3229
	Pa	936	0.1546	0.4044	0.3823
	Sp	837	0.2206	0.1571	1.4045
	Me	927	0.1205	0.4037	0.2985
	Rc	936	0.1231	0.4157	0.2961

**ndhD**	Bc	1491	0.0538	0.4808	0.1120
	Br	1476	0.0541	0.4735	0.1143
	Ci	1500	0.0692	0.5569	0.1243
	Hr	1500	0.0694	0.5457	0.1272
	Vs	1350	0.0572	0.6148	0.0930
	Pa	1497	0.0831	0.4729	0.1757
	Sp	1497	0.0807	0.4983	0.1619
	Me	1497	0.0711	0.4771	0.1491
	Rc	1500	0.0734	0.4417	0.1663
**psaC**	Bc	243	0.0064	0.4508	0.0143
	Br	243	0.0064	0.4508	0.0143
	Ci	243	0.0080	0.5060	0.0158
	Hr	243	0.0080	0.5307	0.0151
	Vs	243	0.0080	0.4811	0.0166
	Pa	243	0.0096	0.5746	0.0167
	Sp	243	0.0096	0.6277	0.0153
	Me	243	0.0080	0.5138	0.0156
	Rc	243	0.0083	0.5655	0.0147
**ndhE**	Bc	303	0.0404	0.3657	0.1107
	Br	303	0.0404	0.3657	0.1107
	Ci	300	0.0474	0.4546	0.1042
	Hr	300	0.0470	0.4063	0.1158
	Vs	303	0.0773	0.5376	0.1438
	Pa	303	0.0885	0.3396	0.2605
	Sp	303	0.0702	0.3893	0.1804
	Me	303	0.0645	0.3642	0.1770
	Rc	303	0.0443	0.3812	0.1163
**ndhG**	Bc	528	0.0610	0.5273	0.1157
	Br	528	0.0610	0.5273	0.1157

	Ci	528	0.0851	0.3536	0.2408
	Hr	528	0.0796	0.3438	0.2315
	Vs	528	0.0617	0.5021	0.1229
	Pa	528	0.0743	0.3524	0.2108
	Sp	528	0.0772	0.3105	0.2486
	Me	528	0.0603	0.4055	0.1487
	Rc	528	0.0722	0.4164	0.1734
**ndhI**	Bc	495	0.0572	0.2813	0.2034
	Br	498	0.0477	0.2961	0.1613
	Ci	483	0.0667	0.2627	0.2541
	Hr	483	0.0733	0.2998	0.2443
	Vs	489	0.0435	0.3384	0.1286
	Pa	498	0.0579	0.2136	0.2709
	Sp	498	0.0576	0.2123	0.2712
	Me	492	1.3905	1.1603	0.8345
	Rc	501	0.0619	0.3132	0.1975
**ndhA**	Bc	537	0.0331	0.4110	0.0805
	Br	1089	0.0492	0.3585	0.1372
	Ci	1074	0.0583	0.4781	0.1219
	Hr	1077	0.0577	0.4788	0.1206
	Vs	1089	0.0532	0.4081	0.1303
	Pa	1086	0.0440	0.3377	0.1303
	Sp	537	0.0444	0.3683	0.1205
	Me	1089	0.0434	0.3034	0.1432
	Rc	1086	0.0412	0.3443	0.1196
**ndhH**	Bc	1179	0.0421	0.5371	0.0784
	Br	1179	0.0421	0.5371	0.0784
	Ci	1179	0.0355	0.6644	0.0534
	Hr	1179	0.0348	0.7576	0.0459

	Vs	1179	0.0421	0.6390	0.0659
	Pa	1179	0.0397	0.5298	0.0749
	Sp	1179	0.0417	0.5311	0.0784
	Me	1179	0.0368	0.5447	0.0675
	Rc	1170	0.0297	0.4765	0.0623
**rps15**	Bc	261	0.1319	0.6877	0.1919
	Br	261	0.1370	0.7108	0.1928
	Ci	261	0.1576	0.9524	0.1655
	Hr	258	0.1544	0.8528	0.0001
	Vs	258	0.2009	0.7836	0.0001
	Pa	261	0.1494	0.8518	0.0001
	Sp	258	0.1482	0.8000	0.1852
	Me	261	0.1079	0.7280	0.1482
	Rc	261	0.0985	0.5976	0.1648

Table S3. Number of simple sequence repeats (SSR) loci in the chloroplast genome of *Byrsonima coccolobifolia* and *B. crassifolia*

		Number of repeats														Total
	Motif	16	15	14	13	12	11	10	9	8	7	6	5	4	3	
Byrsonima coccolobifolia	A/T	1	2	6	3	12	20	34	44	57	117	-	-	-	-	296
	C/G	-	-	-	-	1	3	2	1	4	12	-	-	-	-	23
	AG/AT	-	-	-	-	-	-	-	-	-	-	2	3	20	-	25
	CA/CT	-	-	-	-	-	-	-	-	-	-	-	-	2	-	2
	GA	-	-	-	-	-	-	-	-	-	-	-	-	6	-	6
	TA/TC	-	-	-	-	-	-	-	-	-	1	1	2	7	-	11
	AAC/AAG/AAT/ACG/AGA/AGC/AGT/ATA/ATT	-	-	-	-	-	-	-	-	-	-	-	-	3	18	21
	CAA/CAG/CTG/CTT	-	-	-	-	-	-	-	-	-	-	-	-	-	6	6
	GAA/GAG/GAT/GCA/GCT/GGA/GGT	-	-	-	-	-	-	-	-	-	-	-	-	1	8	9
	TAA/TAG/TAT/TCC/TCT/TGC/TTA/TTC/TTG	-	-	-	-	-	-	-	-	-	-	-	-	-	22	22
	AAGA	-	-	-	-	-	-	-	-	-	-	-	-	-	1	1
	GATT	-	-	-	-	-	-	-	-	-	-	-	-	-	1	1
	TGAT	-	-	-	-	-	-	-	-	-	-	-	-	-	1	1
	TTAA	-	-	-	-	-	-	-	-	-	-	-	-	-	1	1
	ATATA	-	-	-	-	-	-	-	-	-	-	-	-	-	1	1
	ATTAA	-	-	-	-	-	-	-	-	-	-	-	-	-	-	0
	TTATA	-	-	-	-	-	-	-	-	-	-	-	-	-	1	1
															Total	427
Byrsonima crassifolia	A/T	-	-	2	9	9	23	34	37	58	115	-	-	-	-	287
	C/G	-	-	-	-	1	2	1	6	2	13	-	-	-	-	25
	AG/AT	-	-	-	-	-	-	-	-	-	-	2	4	20	-	26
	CA/CT	-	-	-	-	-	-	-	-	-	-	-	-	2	-	2
	GA	-	-	-	-	-	-	-	-	-	-	-	-	6	-	6
	TA/TC	-	-	-	-	-	-	-	-	-	1	1	1	6	-	9
	AAC/AAG/AAT/ACG/AGA/AGC/AGT/ATA/ATT	-	-	-	-	-	-	-	-	-	-	-	-	1	17	18
	CAA/CAG/CTG/CTT	-	-	-	-	-	-	-	-	-	-	-	-	-	6	6
	GAA/GAG/GAT/GCA/GCT/GGA/GGT	-	-	-	-	-	-	-	-	-	-	-	-	1	8	9
	TAA/TAG/TAT/TCC/TCT/TGC/TTA/TTC/TTG	-	-	-	-	-	-	-	-	-	-	-	-	-	20	20
	AAGA	-	-	-	-	-	-	-	-	-	-	-	-	-	1	1
	GATT	-	-	-	-	-	-	-	-	-	-	-	-	-	1	1
	TGAT	-	-	-	-	-	-	-	-	-	-	-	-	-	1	1
	TTAA	-	-	-	-	-	-	-	-	-	-	-	-	-	1	1
	ATATA	-	-	-	-	-	-	-	-	-	-	-	-	-	-	0
	ATTAA	-	-	-	-	-	-	-	-	-	-	-	-	-	1	1
	TTATA	-	-	-	-	-	-	-	-	-	-	-	-	-	1	1
															Total	414

Table S4. Species used in the study and their respective botanic families and accession numbers

Family	Species	Abbreviation	Acession number
Euphorbiaceae	*Manihot esculenta* Crantz	Me	NC_010433
	*Ricinus communis*L.	Rc	NC_016736
Chrysobalanaceae	*Chrysobalanus icaco*L.	Ci	NC_024061
	Hirtella racemosa Lam.	Hr	NC_024060
Malpighiaceae	*Byrsonima cocolobifolia Kunth*	Bc	-
	Byrsonima crassifolia(L.) Kunth	Br	-
Salicaceae	*Populus alba* L.	Pa	NC_008235
	Salix purpurea L.	Sp	NC_026722
Violaceae	*Viola seoulensis* Nakai	Vs	NC_026986
